# Estrogen-induced chromatin looping changes identify a subset of functional regulatory elements

**DOI:** 10.1101/2024.06.12.598690

**Published:** 2024-06-14

**Authors:** Hosiana Abewe, Alexandra Richey, Jeffery M Vahrenkamp, Matthew Ginley-Hidinger, Craig M Rush, Noel Kitchen, Xiaoyang Zhang, Jason Gertz

**Affiliations:** 1 Huntsman Cancer Institute, University of Utah, Salt Lake City, UT 84112, USA; 2 Department of Oncological Sciences, University of Utah, Salt Lake City, UT 84112, USA; 3 Department of Biomedical Engineering, University of Utah, Salt Lake City, UT 84112, USA

## Abstract

Transcriptional enhancers can regulate individual or multiple genes through long-range three-dimensional (3D) genome interactions, and these interactions are commonly altered in cancer. Yet, the functional relationship between changes in 3D interactions associated with regulatory regions and differential gene expression appears context-dependent. In this study, we used HiChiP to capture changes in 3D genome interactions between active regulatory regions of endometrial cancer cells in response to estrogen treatment and uncovered significant differential long-range interactions that are strongly enriched for estrogen receptor α (ER) bound sites (ERBS). The ERBS anchoring differential loops with either a gene’s promoter or distal regions were correlated with larger transcriptional responses to estrogen compared to ERBS not involved in differential interactions. To functionally test this observation, CRISPR-based Enhancer-i was used to deactivate specific ERBS, which revealed a wide range of effects on the transcriptional response to estrogen. However, these effects are only subtly and not significantly stronger for ERBS in differential loops. In addition, we observed an enrichment of 3D interactions between the promoters of estrogen up-regulated genes and found that looped promoters can work together cooperatively. Overall, our work suggests that changes in 3D genome structure upon estrogen treatment identify some functionally important regulatory regions; however, these changes aren’t required for a transcriptional response to E2 in endometrial cancer cells.

## Introduction

Enhancers are critical *cis*-regulatory elements in metazoan genomes that increase the transcription of target genes in response to intrinsic and external signals. A typical human gene is associated with multiple enhancers, each bound by specific transcription factors ^1,2^. These enhancers can reside far from their target gene promoters and interact through long-range chromatin looping events as part of the three-dimensional (3D) genome structure ^3^. Aberrant 3D genome interactions, particularly involving enhancer-promoter contacts, can lead to dysregulation of oncogenes and tumor suppressors ^4^. In fact, most changes in 3D genome structure have been reported in relatively small-scale chromatin loops that have a size range of 5 −100kb and primarily involve enhancer-promoter and enhancer-enhancer interactions ^5–9^. Furthermore, these 3D alterations are seen across different types of cancer ^10–13^ with cancer and normal cells exhibiting remarkably different 3D genome structures ^14–16^.

While looping interactions are a common feature of active enhancers, changes in the 3D genome structure aren’t always associated with differential gene expression. Some studies have detected significant effects on gene regulation that accompany large changes to 3D genome organization ^17–21^. In contrast, other studies have demonstrated that broad rewiring of 3D genome interactions, due to acute depletion of topologically associating domain (TAD) boundaries or TAD architectural proteins (e.g., CTCF or cohesin), were unexpectedly accompanied by modest effects on gene expression ^22–25^. These seemingly contradictory results suggest that changes to the 3D genome impact gene expression to varying degrees depending on context. This inconsistency is also seen in cancer, where abnormal spatial chromatin contacts in association with an active oncogene do not necessarily correlate with changes in gene expression ^26^. To better understand the functional relationship between chromatin-looping interactions and gene expression in response to oncogene activation in cancer cells, we focused on changes in 3D genome structure and the transcriptional response to estrogen receptor α (ER) activation in endometrial cancer cells.

ER is an oncogenic transcription factor in breast and endometrial cancer that is activated by binding to estrogens, such as endogenous 17β-estradiol (E2) ^27–29^. ER binds mostly to distal enhancers and primarily asserts its effects on transcription from a distance through chromatin interactions ^30^. Previous studies have revealed that activation of ER upon estrogen exposure is associated with global reorganization of 3D genome interactions in breast cancer cell lines ^31^. Additionally, a strong correlation between ER and changes in looping interactions has been reported in both breast ^30,32^ and endometrial ^33^ cancer cells, suggesting that ER may play a role in mediating 3D genome structure alterations in response to estrogen treatment, or that it may utilize changes in chromatin looping to impact transcription. However, it remains unclear whether 3D genome changes relate to the functional importance of ER-bound enhancers in terms of the transcriptional response to estrogen exposure. Moreover, the studies into estrogen and 3D genome changes have been focused mainly on breast cancer, while its effect in endometrial cancer, where we have previously shown that ER regulates different genes than in breast cancer ^34^, is poorly understood.

Here, we use HiChIP, a high-resolution technique for analyzing 3D genome structure ^35^, in endometrial cancer cells in response to E2 treatment. We found that long-range interactions that significantly changed upon estrogen exposure were associated with ER-bound sites (ERBS) and highly correlated with increased transcriptional responses to E2. To determine the functional importance of specific interactions on gene regulation, we used CRISPR-based Enhancer-i ^36^ to block ER-bound enhancers from different classes of chromatin interactions or promoters that are part of an E2 upregulated gene cluster. Our results revealed that ERBS involved in chromatin looping are likely to be important for an E2 transcriptional response, regardless of whether their interactions were impacted by E2 treatment, and also uncovered a cooperative relationship between clustered E2 upregulated promoters.

## Results

### The 3D genome structure of endometrial cancer cells is altered in response to estrogen exposure

**Figure 1.**
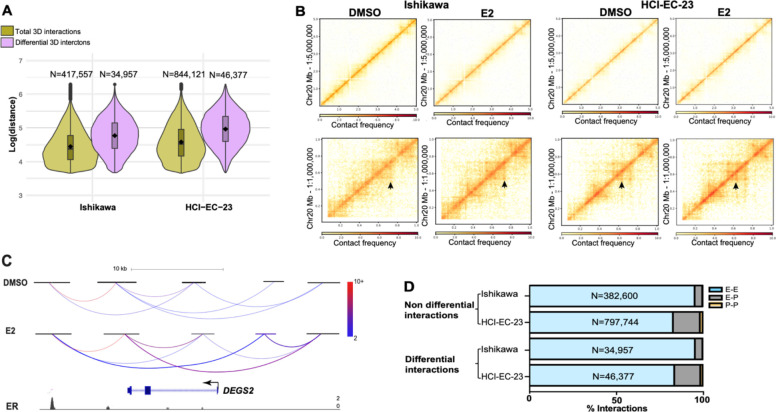
3D genome structure changes upon estrogen induction. A) Violin plots show total 3D genome interactions (green) and differential interactions (purple) in Ishikawa (left) and HCI-EC-23 (right) cell lines identified by HiChIP. B) Heatmaps exhibit chromosome 20 HiChIP signal for DMSO and E2 samples in Ishikawa (left) and HCI-EC-23 (right) cell lines (top panel represents low resolution; bottom panel displays high resolution). C) HiChIP 3D genome interactions at the *DEGS2* locus are shown where the color of the loops represents the normalized read depth and the two differential chromatin loops are shown with thickness. ChIP-seq browser track of ER upon E2 treatment is displayed at the bottom. D) Genomic distribution of non-differential and differential 3D genome interactions in Ishikawa and HCI-EC-23 cell lines are shown.

To investigate the effects of ER activation on the 3D genome structure of endometrial cancer cells, we performed HiChIP in the context of 1-hour treatment with E2 or vehicle. To focus our analysis on active regulatory regions, including enhancers and promoters, we used an antibody against acetylation of lysine 27 of histone H3 (H3K27ac). These experiments were performed in two different ER expressing endometrial cancer cell lines, Ishikawa and HCI-EC-23 ^37^. The metrics for all libraries indicated high-quality data (Figures S1A,B). We identified 417,556 and 844,121 total 3D interactions in Ishikawa and HCI-EC-23 cell lines, respectively, and most of these interactions represented chromatin loops less than 100 kb apart in the linear genome with the median interaction distance being 24.0 kb (Ishikawa) and 33.6 kb (HCI-EC-23) (Figure 1A).

Differential analysis of HiChIP data identified 34,956 and 46,378 3D genome interactions that were impacted by E2 treatment (FDR < 0.05) in Ishikawa and HCI-EC-23 cell lines, respectively. Consistent with previous findings of E2 treatment in breast cancer cells ^37^, the majority of identified differential interactions were longer than non-differential loops, representing long-range chromatin looping events (median size: 59.0 kb in Ishikawa and 94.0 kb in HCI-EC-23; Figure 1A), implying substantial involvement of distal enhancers in altering the 3D genome structure upon estrogen treatment. Taking chromosome 20 as an example, at low resolution the 3D genome structure did not change dramatically but changed significantly at high resolution upon estrogen treatment (Figure 1B). Most of these observed changes involved increased interactions with E2 treatment rather than reduced interactions (68.8% and 69.9% of differential loops increase in Ishikawa and HCI-EC-23, respectively). As an example, *DEGS2* is an E2 regulated gene that exhibited an increase in interaction frequencies with distal enhancers bound by ER upon estrogen treatment (Figure 1C). Interactions involving two distal regulatory regions (E-E) predominated over enhancer–promoter (E-P) or promoter-promoter (P-P) interactions (Figure 1D), implying the involvement of distal enhancers in altering the 3D genome structure upon E2 induction. Importantly, H3K27ac signal at peaks defined by self-ligation products was only impacted by the 1-hour E2 treatment at 1 region in Ishikawa cells and 2 regions in HCI-EC-23 cells, indicating that changes in H3K27ac are not responsible for the observed differential chromatin loops. Overall, this data provided a foundation for studying the relationship between chromatin looping and transcriptional responses to estrogen in endometrial cancer cells.

### Differential 3D interactions are associated with the estrogen transcriptional response

**Figure 2.**
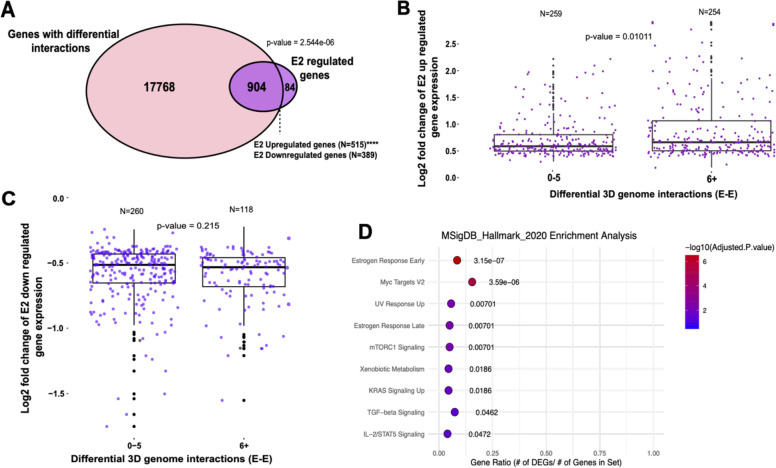
3D genome structure changes upon estrogen treatment associate with differential gene expression. A) Overlap is shown between E2 regulated genes (purple) and those involved with differential 3D interactions (pink) in Ishikawa cells; ****p < 0.00001. (B-C) Boxplots demonstrate relative expression of E2 up-regulated (B) down-regulated (C) genes associated with less than 6 differential 3D genome interactions and more than 5 differential 3D genome interactions in Ishikawa cells. D) The rank of hallmark gene sets associated with E2 regulated genes with differential 3D genome interactions in Ishikawa cells is shown.

To understand how changes in 3D genome interactions in response to E2 treatment relate to gene expression, we first annotated differential 3D genome interactions that have anchors within 5kb of transcription start sites (TSS). We identified 6027 and 7755 genes that harbor differential interactions in response to E2 induction in Ishikawa and HCI-EC-23 cell lines, respectively. However, as the majority of differential interactions involve two distal regulatory regions, we also annotated differential chromatin interactions that have anchors withhin 100kb of TSS and found 18,672 and 19,746 genes associated with differential 3D interactions in response to E2 induction in Ishikawa and HCI-EC-23 cell lines, respectively. There was a significant overlap of genes in differential interactions within 5kb (36.5%; p-value = 2.0e-19, hypergeometric distribution) or 100kb (92.3%; p-value = 7.86e-488, hypergeometric distribution) of TSS between the two cell lines, suggesting that the endometrial cancer models consistently respond to E2 treatment in terms of genome organization (Figures S1C,D). Estrogen induction has been previously shown to impact the expression of hundreds of genes in both endometrial cancer cell lines ^38,39^, particularly in Ishikawa. Integration of E2 responsive genes with HiChIP data revealed that Ishikawa cells exhibited a significant enrichment of E2 regulated genes, particularly up-regulated genes, in those involved with differential 3D interactions (Figure 2A; p-value = 2.54e-06, hypergeometric distribution). In contrast, HCI-EC-23 cells showed a weaker association between E2 regulated genes and differential 3D genome interactions (Figure S2A; p-value =0.21, hypergeometric distribution), which might be due to a lower number of E2 responsive genes in HCI-EC-23.

We next determined whether differential 3D interactions were predictive of larger transcriptional responses to E2. Using linear regression models, we found that more differential E-E interactions were associated with larger up-regulated (Figure 2B; p-value = 0.01011, Wilcoxon rank sum test) rather than down-regulated (Figure 2C; p-value = 0.215, Wilcoxon rank sum test) transcriptional changes to E2 treatment in Ishikawa cells; however, HCI-EC-23 did not show a significant association with either up-regulation (Figure S2C; p-value = 0.5032, Wilcoxon rank sum test) or down-regulation (Figure S2D; p-value = 0.1731, Wilcoxon rank sum test) in response to E2 treatment. Gene ontology analysis indicated that the genes that change expression and 3D interactions in response to E2 induction are strongly associated with several biological signaling pathways previously connected to E2 treatment, including estrogen and MYC signaling pathways in both Ishikawa (Figure 2D) and HCI-EC-23 cells (Figure S2E). These results suggest that chromatin looping changes in response to estrogen treatment are associated with differentially expressed genes in Ishikawa cells, but to a lesser extent in HCI-EC-23, hence most subsequent studies involving E2 response were conducted in Ishikawa cells. Additionally, these results reveal several other genes that exhibit 3D looping changes or gene expression changes, but not both.

### ER binding events are enriched in differential 3D interactions

**Figure 3.**
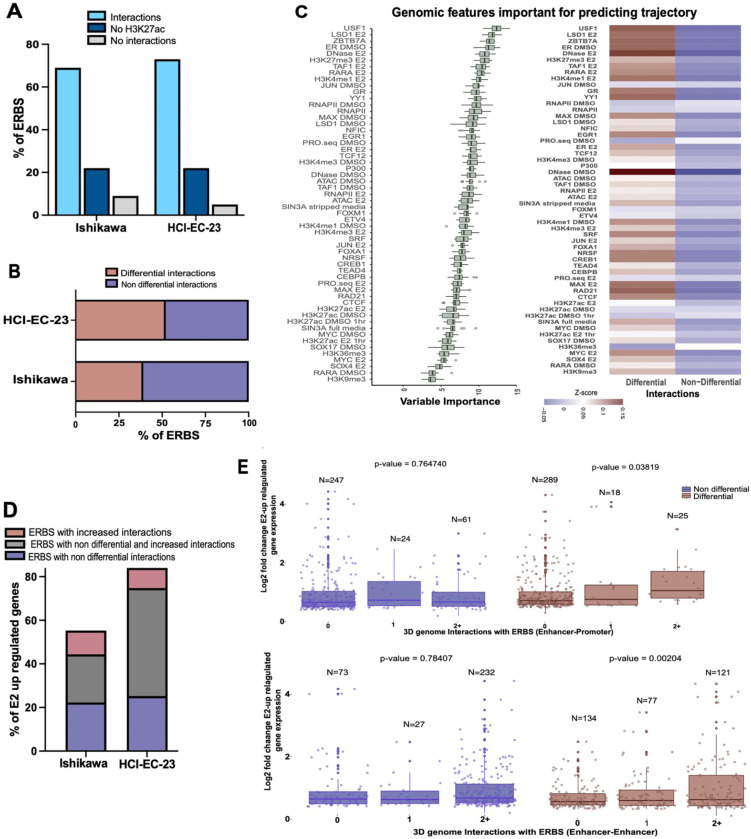
ERBS exhibit distinctive features when involved in differential 3D interactions. (A-B) Bar graphs portray ERBS associated with 3D genome interactions and their classifications in endometrial cell lines. (C, left) Ranking of genomic features based on their importance for ERBS associated with differential or non-differential interactions is shown. (C, right) Heatmap displays the average signal intensity for top-ranked genomic features clustered by ERBS with differential or non-differential interactions. (D) Bar graph shows the classification of E2 up-regulated genes associated with ERBS in (non) differential interactions within 100 kb of their TSS. (E) Box plots compare fold change for E2 up-regulated genes split by the number of non-differential (left, blue) or differential (right, brown) enhancer-promoter (top) or enhancer-enhancer (bottom) loops involving ERBS.

Since ER is the main mediator of the transcriptional response to estrogen in endometrial cancer cells, we next evaluated the relationship between ER genomic binding and chromatin looping. Integration of previously collected ER ChIP-seq data ^38,39^ with the HiChIP data revealed that the majority of ERBS were found in 3D genome interactions (69% in Ishikawa and 70% in HCI-EC-23) (Figure 3A). Of the ERBS not found to be in 3D genome interactions, approximately 22% of ERBS did not harbor H3K27ac and, therefore, 3D genome interactions involving these sites might have been missed for technical reasons, while less than 10% of ERBS that had detectable H3K27ac signal were not found in detectable 3D interactions (figure 3A).

We next compared ERBS with changes in 3D genome interactions caused by E2 treatment. We observed a very strong enrichment of ERBS in differential 3D genome interactions (Figure 3B, p-value = 2.46e-37, hypergeometric distribution) with close to half of ERBS serving as anchors for differential 3D genome interactions. The majority of these ERBS (71.9 % in Ishikawa and 79.2 % in HCI-EC-23) were associated with gained 3D interactions. To understand the differences between ERBS associated with differential or non-differential interactions, we used Boruta, a random forest feature selection approach to look for genomic features in Ishikawa cells that distinguish ERBS anchoring differential 3D interactions ^40^. Several transcription factors as well as chromatin accessibility were enriched at ERBS with differential 3D interactions, while ERBS associated with non-differential interactions exhibited features of actively transcribed regions, including RNA polymerase II binding, PRO-seq signal, and H3K36me3 (Figure 3C). These results suggest that ERBS associated with non-differential interactions were already engaged in transcription prior to E2 treatment while ERBS associated with differential 3D genome interactions are open regulatory regions with less active transcription.

To determine how ERBS-associated changes in 3D genome interactions relate to the E2 transcriptional response, we first identified ERBS associated with E2 up-regulated genes in Ishikawa cells and then split ERBS based on whether they served as anchors for differential, non-differential, or both types of 3D interactions. We found that differential and non-differential ERBS containing loops were similarly enriched for E2 up-regulated genes (Figure 3D). In addition, ERBS with differential or non-differential interactions were equally likely to regulate genes that respond early or late during an 8-hour time course ^40^ (p = 0.55, Fisher’s Exact Test). We next used linear regression to determine if ERBS associated with differential 3D interactions were predictive of larger transcriptional responses to E2. We observed that the size of the transcriptional response to E2 was positively correlated with the number of promoter-distal or distal–distal differential 3D interactions anchored by ERBS, but not with the number of promoter-distal or distal–distal non-differential interactions anchored by ERBS (Figure 3E). Overall, these findings indicate that ER genomic binding sites are equally likely to be involved with non-differential or differential chromatin loops and that ERBS in differential 3D interactions are more open and less transcriptionally active prior to E2 treatment, but associate with larger changes in the expression of their target genes upon E2 induction.

While differential 3D interactions were highly enriched with ERBS upon E2 treatment, most differential 3D interactions were not associated with ER in Ishikawa (89%) and HCI-EC-23 (90%) cells. To identify the features of differential interactions that didn’t exhibit ER binding, we first classified non-differential or differential 3D interactions in both cell lines based on whether a loop anchor overlapped an ERBS (ER primary interaction) or whether a loop anchor interacted with an ERBS through another loop (ER secondary interaction); the remaining interactions were categorized as ER-unrelated interactions (Figure S3A and S3B). We used Boruta analysis to identify features that distinguish ER-unrelated differential 3D interactions from ER-unrelated non-differential interactions in Ishikawa cells. The results showed that ER-unrelated differential interactions are enriched for several transcription factors and chromatin accessibility (Figure S3C). When we further categorized ER-unrelated differential interactions based on whether they increased or decreased, we found that decreased ER-unrelated 3D interactions exhibited a stronger enrichment of transcription factors than increased 3D interactions. On the contrary, increased 3D interactions without ERBS were uniquely enriched with features of active transcription, including RNA polymerase II binding and H3K36me3 (Figure S3D). While the question remains as to why thousands of non-ER associated loops are changing in interaction frequency, the strongest pattern that we observed is a reduction in looping at anchors with a high level of transcription factor binding and chromatin accessibility, which may indicate a competition for activating regulatory events when ER is active.

### ERBS in differential chromatin loops are slightly more likely to regulate transcription

The analysis described above led us to hypothesize that ERBS with differential 3D interactions due to E2 treatment are more likely to impact transcription. To investigate the functional importance of different types of ERBS, we used the CRISPR-based Enhancer-i system ^41^ to repress the activity of individual ERBS and measure the gene expression response of target genes to an 8-hour E2 induction. We classified ERBS first into differential or non-differential 3D genome interactions and further classified the sites into candidate enhancer-promoter (E-P) or candidate enhancer-enhancer (E-E) interactions, targeting at least 5 ERBS in each category. Results from our previously published Enhancer-i study ^36^ were combined with experiments performed for this study.

**Figure 4.**
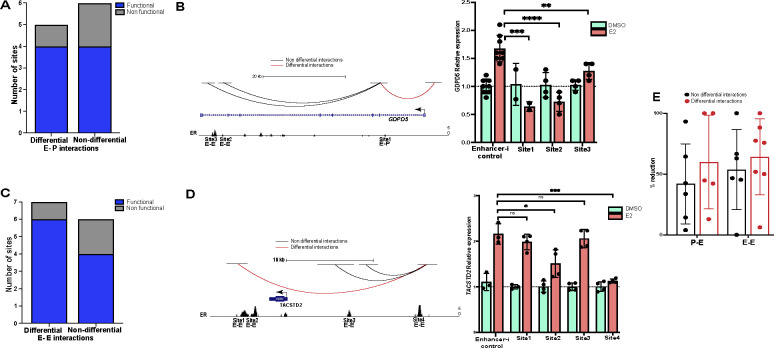
ERBS in differential chromatin loops are subtly more important for the transcriptional response to estrogen. (A/C) The functionality of ERBS based on Enhancer-i that are anchors of differential and non-differential E-P (A) or E-E (C) interactions is shown. (B/D, left) Genome browser tracks of *GDPD5* (B) and *TACSTD2* (D) show differential (red) and non-differential (black) 3D genome interactions as well as ER ChIP-seq signal. (B/D, right) Relative expression of *GDPD5* (B) and *TACSTD2* (D) normalized to DMSO when a candidate ERBS is targeted with Enhancer-i upon DMSO (green) or E2 (orange) treatment is displayed. Error bars represent the SEM; ****p < 0.00001, ***p < 0.001, **p < 0.01 and *p < 0.05, unpaired t-test, while ns depicts statistical insignificance. (E) Percent reduction is shown for each class of ERBS associated with different types of 3D interactions. Each dot represents an ERBS.

In the E-P category, 80% of ERBS with increased differential interactions and 67% of ERBS with non-differential interactions were found to be functionally important for the E2 transcriptional response (Figures 4A and S4A). In the E-E category, 86% of ERBS with increased differential interactions and 67% of ERBS with only non-differential interactions were found to be functional (Figure 4C and S4A). For example, there are 3 ERBS that are near *GDPD5*, an E2 up-regulated gene. ERBS 1, which anchors a differential increased E-P interaction, significantly impacts the expression of *GDPD5* (Figure 4B). ERBS 2 and 3 near *GDPD5* anchor non-differential E-E interactions that significantly impact *GDPD5* expression (Figure 4B). Another example is the 4 ERBS near *TACSTD2*, an E2 up-regulated gene. ERBS 1, 2, and 4 anchor differential increased E-E interactions, and ERBS 2 and 4 significantly impact the expression of *TACSTD2*, whereas ERBS 1 doesn’t significantly impact the expression of *TACSTD2*. ERBS 3 anchors non-differential E-E interactions and does not significantly impact *TACSTD2* expression (Figure 4D). The successful targeting of each ERBS by Enhancer-i was validated using ChIP-seq (Figure S5A). Overall, 83% of ERBS in differential increased 3D interactions and 67% of ERBS in non-differential chromatin loops demonstrated a significant impact on the E2 transcriptional response; however, this difference was not significant with the 24 ERBS tested (p-value =0.4995; Fisher’s exact test). In addition, we examined the transcriptional effect of each ERBS and found that ERBS that anchor differential increased interactions reduced the E2 response by an average of 62% whereas ERBS that do not anchor differential interactions lead to an average reduction of 48% when targeted by Enhancer-i (Figure 4E), which was not statically significant (p-value = 0.5037, t-test). These results suggest that ERBS associated with differential increased interactions trend toward a higher likelihood of being functional and causing larger effects on transcription. At the same time, the difference is subtle, and several ERBS that only anchor non-differential chromatin loops contribute to the transcriptional response to estrogen.

### E2 up-regulated gene promoters are likely to reside in physical hubs that can cooperate in an estrogen response

**Figure 5.**
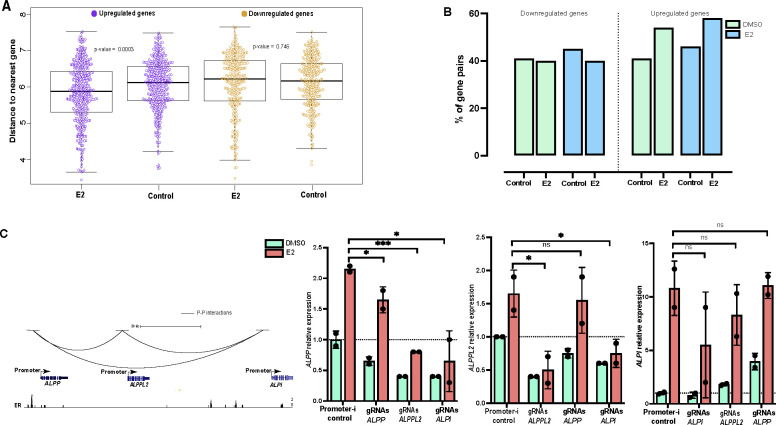
E2 up-regulated gene promoters are enriched in physical interactions that can mediate their transcriptional response to estrogen. (A) The plot displays the genomic distances among E2 responsive genes or randomly selected control genes (upregulated genes (purple) and downregulated genes (yellow)). (B) Bar plot shows the percentage of E2 regulated or control gene pairs within 150 kb that have promoter-promoter interactions (left, downregulated genes; right, upregulated genes) upon DMSO (green) or E2 (blue) treatment. (C, left) Genome browser tracks of *ALPP-ALPPL2-ALPI* gene cluster displaying P-P interactions and ER ChIP-seq signal. (C, right) Relative expression with DMSO (green) or E2 (orange) treatment of each gene when the genes’ promoters in the cluster are targeted by Enhancer-i is displayed. Error bars represent the SEM; ****p < 0.00001, ***p < 0.001, **p < 0.01 and *p < 0.05, unpaired t-test, while ns depicts statistical insignificance.

In addition to E-P and E-E interactions discussed above, our analysis revealed several promoter-promoter (P-P) interactions in the HiChIP data. When analyzing the promoters of estrogen-induced genes, we initially looked at promoter proximity in the linear genome. Evaluation of the distance between promoters showed that E2 up-regulated genes cluster together in the genome more than expected by chance (p-value = 0.00031, Wilcoxon rank sum test) (Figure 5A), indicating that some genes may be co-regulated by E2. Using a 150 kb distance cutoff for potentially co-regulated gene pairs, we identified 81 E2 up-regulated and 49 E2 down-regulated gene pairs. Comparison to HiChIP data revealed that many of the paired E2-regulated gene promoters physically interacted with each other, where more promoter interactions were observed within E2 up-regulated gene pairs, both with and without E2 treatment, compared to randomly selected gene pairs within 150 kb (Figure 5B). These observations suggest that many E2 upregulated genes reside in pre-existing promoter interaction hubs, which raises an interesting question as to whether a functional relationship exists between these interacting promoters in terms of their transcriptional response to estrogen.

To explore the functional relationship between interacting promoters, we targeted interacting promoters of an E2 up-regulated gene cluster (*ALPP-ALPPL2-ALPI*) using Enhancer-i and determined how the activity of one gene in the cluster impacted other cluster genes. The *ALPP-ALPPL2-ALPI* gene cluster exhibited cooperative gene expression, where targeting a single promoter not only affected the targeted gene’s expression but also the expression of other genes in the cluster (Figure 5C). However, the cooperation was not equal; *ALPI* had the most important promoter in the group as it impacted all genes, *ALPPL2*’s promoter cooperated with *ALPP*, and *ALPP*’s promoter was only important for its own expression. The successful targeting of each promoter by Enhancer-i was validated using ChIP-seq (Figure S5B). Overall, these results revealed that E2 up-regulated gene promoters can reside in physical hubs and potentially cooperate in the transcriptional response to estrogen.

## Discussion

The relationship between 3D genomic structure and gene expression is complex and has been described as context-specific by several studies ^42,43^. Some studies have reported minimal changes in expression when large perturbations in genome architecture are observed, while other studies have found large changes in expression for particular genes when the genome structure is altered. In this study, we functionally dissect the relationship between changes in the 3D genome structure and gene regulation in the context of estrogen signaling in endometrial cancer cells. Our focus is on estrogen signaling for several reasons including endogenous activation by estrogen in endometrial cancer cells, ER activation through estrogen signaling is a key oncogenic event in endometrial cancer ^27^, and ER is a well-studied transcription factor, which provides a strong foundation for interpretation of results. We found that 1 hour of estrogen treatment was sufficient to significantly impact tens of thousands of 3D genome interactions. The observed changes were enriched at genes that change expression; however, many genes harbor differential chromatin loops without concomitant changes in gene expression. This observation could be caused by changes in 3D interactions that are unrelated or at least non-productive concerning gene regulation, or the non-responsive genes could be unresponsive to an 8-hour E2 treatment and may require other stimuli or a different amount of time to respond.

Since ER is the main mediator of transcriptional effects of estrogen in these endometrial cancer models, we analyzed 3D genomic interactions in the context of ER genomic binding. Most ERBS served as anchors for chromatin loops, and approximately half of ERBS anchored loops increase in frequency when cells are treated with estrogen for 1 hour. Further analysis uncovered a relationship between ERBS with differential 3D interactions having more accessible chromatin, several transcription factors bound, and less active transcription based on PRO-seq and RNA polymerase II binding. This observation is consistent with sites of active transcription exhibiting higher motility ^44^ and findings in breast cancer cells that estrogen-dependent enhancers increase contact frequencies with their promoters in a transcription-independent manner ^32^. Our findings, together with previous studies, suggest a model in which ER binding does not impact 3D interaction frequencies at regulatory regions that are already transcriptionally active and already highly mobile, while ER binding to transcriptionally inactive sites with accessible chromatin is more likely to increase looping frequencies.

Despite a strong enrichment of ERBS at differential chromatin loops, the majority of differential 3D interactions were not connected to ER in both cell lines. Further analysis uncovered that ER-unrelated differential 3D interactions were anchored by several transcription factors and more open chromatin than ER-unrelated non-differential interactions. The majority of decreased 3D interactions were linked to several transcription factors other than ER and highly associated with less transcription, while the increased 3D interactions without ER were associated with active transcription. These results were consistent with previous ER-positive breast cancer findings, where 3D differential interactions that changed upon estrogen were predominantly associated with transcription factors, such as CTCF ^45,46^, which was similarly enriched with decreased ER-unrelated 3D interactions in this study. Overall, these findings reveal that changes in 3D genome interactions upon E2 treatment are potentially the result of a combined effort or competition between ER and several other genomic features, yet further studies are needed to understand the mechanisms underlying these ER-unrelated changes.

Through correlative analysis, we found that increases in 3D interaction frequency were associated with larger transcriptional responses to estrogen. This finding led us to hypothesize that ERBS with changes in looping frequency have larger expression effects on their target genes. To test this hypothesis, we took a functional approach by targeting different sets of ERBS with Enhancer-i. In total, we analyzed 24 ERBS split into groups of differential or non-differential 3D interactions as well as enhancer-promoter or enhancer-enhancer interactions. The overall patterns that we observed were very similar between ERBS involved in enhancer-promoter and enhancer-enhancer interactions, suggesting that enhancer-enhancer 3D contacts are likely important for the ability of ERBS to regulate gene expression. Across both types of contacts, we found that ERBS that anchor differential 3D interactions functionally impact the transcriptional response to estrogen at a higher percentage than ERBS that only anchor constant 3D interactions. However, this difference was not significant, possibly due to our study being underpowered to detect a subtle difference in functionality between these types of ERBS. While differential chromatin looping upon E2 treatment may lead to larger changes in the transcriptional response, it is clear that ERBS without changes in 3D interactions plays an important role in transcription regulation.

In addition to chromatin loops that involve enhancers, we also observed several promoter-promoter 3D interactions between genes that are up-regulated by estrogen. This finding goes along with our observation that estrogen upregulated genes cluster in close proximity within the genome more than expected by chance. Previous studies on chromatin interactions associated with RNA polymerase II have revealed that most genes with promoter-promoter interactions are transcribed cooperatively ^47,48^, while other findings have detected transcriptional competition among neighboring genes ^49^. To determine how 3D connected promoters of E2 up-regulated genes impact one another, we used Enhancer-i to target a gene’s promoter and analyzed how other genes with connected promoters respond to estrogen. A cooperative relationship was observed among the three E2 up-regulated genes, whose promoters physically interacted in 3D space; however, the promoter strength was not equal among these genes. This study only surveyed 3D interactions of one group of E2 regulated genes, therefore competition and other complex relationships between the promoters of E2 regulated genes may exist within different gene clusters.

## Methods

### Cell culture

Ishikawa and HCI-EC-23 cells were grown in full media: RPMI 1640 medium (ThermoFisher) supplemented with 10% fetal bovine serum (FBS) (ThermoFisher) and 1% penicillin-streptomycin (ThermoFisher) and were incubated at 37°C with 5% CO_2_. Cells were transferred and maintained in hormone-depleted media consisting of phenol red-free RPMI 1640 (ThermoFisher) supplemented with 10% charcoal-dextran FBS (Sigma Aldrich) and 1% penicillin-streptomycin (ThermoFisher) for 5 days prior to E2 treatment.

### HiChIP

The 3D genome structure was analyzed using the published HiChIP protocol ^35^, which consists of three main phases, including in situ contact generation, sonication, and chromatin immunoprecipitation. In brief, crosslinked nuclei were extracted from 10 million cells treated with either 10 nM E2 or DMSO for 1 hour and digested with the restriction enzyme DpnII. The restriction fragment overhangs were filled with biotinylated dATP to mark the DNA ends. Biotin-marked DNA ends were ligated to covalently link interacting regions. Following proximity ligation, the nuclei with *in situ* generated contacts were pelleted and suspended in a nuclear lysis buffer. Crosslinked chromatin was sonicated using an EpiShear probe-in sonicator (Active Motif) with three cycles of 30 seconds at an amplitude of 40% with 30 seconds of rest between cycles. The antibody that recognized H3K27ac (Abcam, ab4729) was used to pull down crosslinked chromatin associated with H3K27ac overnight at 4C. A/G magnetic beads were used for crosslinked chromatin purification. Upon reverse crosslinking, biotin-labeled post-immunoprecipitation DNA was captured by streptavidin C1 magnetic beads (Invitrogen). Tn5 (Illumina) was used to fragment captured biotin-labeled DNA and prepare molecules for PCR generation of sequencing libraries.

HiChIP libraries were sequenced on an Illumina NovaSeq 6000 as paired-end 50 basepair reads to an average depth of 300–400 million read-pairs per sample. Reads were aligned to the human hg19 reference genome using HiC-Pro ^50^ to extract informative unique paired-end tags (PETs). Hichipper ^51^ was used to perform restriction site bias-aware modeling of the output from HiC-Pro and to call interaction loops. These loops were filtered where loops with at least one or more reads in both biological replicates of a particular treatment (E2 or DMSO) were kept and used for downstream analyses. Chromatin interactions that significantly change upon 1 hour of E2 induction were identified using DESeq2 with an adjusted p-value cutoff of 0.05. Enhancer-enhancer loops were assigned to genes using GREAT ^52^ with a cutoff of 100kb. Promoters were defined as any anchor that overlapped within 500bp of the transcription start sites of University of California Santa Cruz (UCSC) Known Genes.

### Enhancer interference

gRNAs targeting regions of interest for Enhancer-i (4 gRNAs per targeted region) were designed using the Benchling gRNA design tool ^53^ (see Table S1 for sequences). gRNAs were cloned into pGL3-U6-PGK-Puro (Addgene 51133, a gift from Xingxu Huang) as previously described ^36^. Ishikawa cells expressing SID4x-dCas9-KRAB fusion protein ^36^ were cultured in full media supplemented with 600 ng/uL G418 and incubated at 37°C with 5% CO_2_. Six days before transfection, cells were transferred and maintained in hormone-depleted media with 600ng/uL G418. 24 hours before transfection, cells were plated in a 24-well plate at 90,000 cells per well and transfected with gRNAs using Fugene HD (Promega) at a manufacturer-suggested 3:1 reagent:DNA ratio. At 36 hours post-transfection, cells were treated with 1 μg/mL puromycin to select for cells successfully transfected with gRNA plasmids. Selected cells were induced 24 hours later with 10nM E2 or DMSO (vehicle) for 8 hours.

### ChIP-seq

Ishikawa cells expressing the SID4x-dCas9-KRAB fusion protein were cultured as described in the transfection method section. 24 hours before transfection, cells were plated in 15cm dishes at approximately 60% confluency. gRNAs targeting 24 different sites were combined into four separate pools; each pool contained gRNAs that targeted 6 different sites nearby different genes and were transfected into plated cells. The remaining steps, including E2 treatment, were conducted as described in the transfection method section. ChIP-seq libraries were generated as previously described ^54^ and the antibody used for this study was FLAG M2 (Sigma-Aldrich F1894). The ChIP-seq libraries were sequenced on an Illumina Novaseq X as paired-end 150 bp. Bowtie ^55^ was used to trim the reads to 50bp by removing 100bp from the 3’ ends and aligning the trimmed reads to the human hg19 reference genome. Samtools view ^56^ was used to convert aligned sequences to bam format; Macs2 ^57^ was used to create bedgraphs. Bedgraphs were converted to bigwig using UCSC’s BedgraphtToBigWig tool and visualized on the UCSC genome browser.

### RNA isolation and qRT-PCR

Cells were lysed in RLT Plus (Qiagen) supplemented with 1% beta-mercaptoethanol (Sigma). RNA was extracted and purified using the ZR-96-well Quick-RNA kit (Zymo Research). Gene expression was quantified using reverse transcription quantitative PCR (RT- qPCR) at 40 cycles on a CFX Connect light cycler (BioRad). qPCR reaction mixtures were set up with reagents from the Power SYBR Green RNA-to-Ct 1-step kit (ThermoFisher) with 25–50ng RNA per reaction and primers are listed in Table S2. Melt curve analysis was conducted to assess amplicon qPCR length and specificity. Relative expression was determined using the ΔΔCt method; the reference gene used for this assay was *CTCF*. The significance of gene expression change between experimental conditions was determined by unpaired t-test with a p-value cutoff of 0.05.

### Analysis of important features

The Boruta analysis was performed as described by Ginley-Hidinger et al. ^40^ including the feature data sets analyzed. For the analysis looking at differences between differential and non-differential loops, each anchor was included separately. Due to disparities in the size of different sets, we down-sampled the larger set and Boruta ^58^ was run using 100 random downsamplings. For linear regression analysis of the association between the numbers of loops and expression responses to E2, data was first compiled in a gene-centric table with each row containing a gene, the log_2_ fold change after E2 treatment, and the sum of loops that interact with that gene in a distal to distal, distal to promoter, and promoter to promoter interaction. We used the lm function in R version 4.3.1 as follows: lm(formula = Log_2_ Fold Change ~ Distal-Distal + Promoter-Distal + Promoter-Promoter). This analysis was performed using all loops or only ERBS anchored loops. Significance was determined using a t-test on the coefficients with a cutoff of 0.05.

## Supplementary Material

Supplement 1

## Data Availability

HiChIP data are available at the Gene Expression Omnibus under accession GSE269670.

## References

[1] Furlong E. E. M. & Levine M. Developmental enhancers and chromosome topology. Science 361, 1341–1345 (2018). 30262496 10.1126/science.aau0320 PMC6986801

[2] Andersson R. An atlas of active enhancers across human cell types and tissues. Nature 507, 455–461 (2014). 24670763 10.1038/nature12787 PMC5215096

[3] Heidari N. Genome-wide map of regulatory interactions in the human genome. Genome Res. 24, 1905–1917 (2014). 25228660 10.1101/gr.176586.114 PMC4248309

[4] Valton A.-L. & Dekker J. TAD disruption as oncogenic driver. Curr. Opin. Genet. Dev. 36, 34–40 (2016). 27111891 10.1016/j.gde.2016.03.008 PMC4880504

[5] Yang M. Proteogenomics and Hi-C reveal transcriptional dysregulation in high hyperdiploid childhood acute lymphoblastic leukemia. Nat. Commun. 10, 1–15 (2019). 30944321 10.1038/s41467-019-09469-3 PMC6447538

[6] Taberlay P. C. Three-dimensional disorganization of the cancer genome occurs coincident with long-range genetic and epigenetic alterations. Genome Res. 26, 719–731 (2016). 27053337 10.1101/gr.201517.115 PMC4889976

[7] Flavahan W. A. Insulator dysfunction and oncogene activation in IDH mutant gliomas. Nature 529, 110–114 (2016). 26700815 10.1038/nature16490 PMC4831574

[8] Braun R. Single Chromosome Aneuploidy Induces Genome-Wide Perturbation of Nuclear Organization and Gene Expression. Neoplasia N. Y. N 21, 401–412 (2019). 10.1016/j.neo.2019.02.003 PMC6434407 30909073

[9] O’Mara T. A., Spurdle A. B. & Glubb D. M. Analysis of Promoter-Associated Chromatin Interactions Reveals Biologically Relevant Candidate Target Genes at Endometrial Cancer Risk Loci. Cancers 11, (2019). 10.3390/cancers11101440 PMC6826789 31561579

[10] Kantidze O. L. The anti-cancer drugs curaxins target spatial genome organization. Nat. Commun. 10, (2019). 10.1038/s41467-019-09500-7 PMC6441033 30926878

[11] Wu P. 3D genome of multiple myeloma reveals spatial genome disorganization associated with copy number variations. Nat. Commun. 8, 1–11 (2017). 29203764 10.1038/s41467-017-01793-w PMC5715138

[12] Hnisz D. Activation of proto-oncogenes by disruption of chromosome neighborhoods. Science 351, 1454–1458 (2016). 26940867 10.1126/science.aad9024 PMC4884612

[13] Barutcu A. R. Chromatin interaction analysis reveals changes in small chromosome and telomere clustering between epithelial and breast cancer cells. Genome Biol. 16, (2015). 10.1186/s13059-015-0768-0 PMC4587679 26415882

[14] Achinger-Kawecka J., Taberlay P. C. & Clark S. J. Alterations in Three-Dimensional Organization of the Cancer Genome and Epigenome. Cold Spring Harb. Symp. Quant. Biol. 81, 41–51 (2016). 28424341 10.1101/sqb.2016.81.031013

[15] Kloetgen A. Dynamic 3D chromosomal landscapes in acute leukemia. bioRxiv 724427 (2019) doi:10.1101/724427.

[16] Li T. Integrative Analysis of Genome, 3D Genome, and Transcriptome Alterations of Clinical Lung Cancer Samples. Genomics Proteomics Bioinformatics 19, 741–753 (2021). 34116262 10.1016/j.gpb.2020.05.007 PMC9170781

[17] Kim K. L. Dissection of a CTCF topological boundary uncovers principles of enhancer-oncogene regulation. Mol. Cell 84, 1365–1376.e7 (2024). 38452764 10.1016/j.molcel.2024.02.007 PMC10997458

[18] Smits W. K. Elevated enhancer-oncogene contacts and higher oncogene expression levels by recurrent CTCF inactivating mutations in acute T cell leukemia. Cell Rep. 42, 112373 (2023). 37060567 10.1016/j.celrep.2023.112373 PMC10750298

[19] Franke M. Formation of new chromatin domains determines pathogenicity of genomic duplications. Nature 538, 265–269 (2016). 27706140 10.1038/nature19800

[20] Lupiáñez D. G. Disruptions of Topological Chromatin Domains Cause Pathogenic Rewiring of Gene-Enhancer Interactions. Cell 161, 1012–1025 (2015). 25959774 10.1016/j.cell.2015.04.004 PMC4791538

[21] Pinoli P., Stamoulakatou E., Nguyen A.-P., Martínez M. R. & Ceri S. Pan-cancer analysis of somatic mutations and epigenetic alterations in insulated neighbourhood boundaries. PLOS ONE 15, e0227180 (2020). 31945090 10.1371/journal.pone.0227180 PMC6964824

[22] Despang A. Functional dissection of the Sox9 – Kcnj2 locus identifies nonessential and instructive roles of TAD architecture. Nat. Genet. 51, 1263–1271 (2019). 31358994 10.1038/s41588-019-0466-z

[23] Schwarzer W. Two independent modes of chromatin organization revealed by cohesin removal. Nature 551, 51–56 (2017). 29094699 10.1038/nature24281 PMC5687303

[24] Nora E. P. Targeted Degradation of CTCF Decouples Local Insulation of Chromosome Domains from Genomic Compartmentalization. Cell 169, 930–944.e22 (2017). 28525758 10.1016/j.cell.2017.05.004 PMC5538188

[25] Rao S. S. P. Cohesin Loss Eliminates All Loop Domains. Cell 171, 305–320.e24 (2017). 28985562 10.1016/j.cell.2017.09.026 PMC5846482

[26] Li Y. Alteration of CTCF-associated chromatin neighborhood inhibits TAL1-driven oncogenic transcription program and leukemogenesis. Nucleic Acids Res. 48, 3119–3133 (2020). 32086528 10.1093/nar/gkaa098 PMC7102946

[27] Rodriguez A. C., Blanchard Z., Maurer K. A. & Gertz J. Estrogen Signaling in Endometrial Cancer: a Key Oncogenic Pathway with Several Open Questions. Horm. Cancer 10, 51–63 (2019). 30712080 10.1007/s12672-019-0358-9 PMC6542701

[28] Shen F., Gao Y., Ding J. & Chen Q. Is the positivity of estrogen receptor or progesterone receptor different between type 1 and type 2 endometrial cancer? Oncotarget 8, 506–511 (2016). 10.18632/oncotarget.13471 PMC5352172 27888807

[29] Zwart W. Oestrogen receptor–co-factor–chromatin specificity in the transcriptional regulation of breast cancer. EMBO J. 30, 4764–4776 (2011). 22002538 10.1038/emboj.2011.368 PMC3243612

[30] Fullwood M. J. An oestrogen-receptor-α-bound human chromatin interactome. Nature 462, 58–64 (2009). 19890323 10.1038/nature08497 PMC2774924

[31] Mourad R. Estrogen Induces Global Reorganization of Chromatin Structure in Human Breast Cancer Cells. PLOS ONE 9, e113354 (2014). 25470140 10.1371/journal.pone.0113354 PMC4255042

[32] Acuña L. I. G., Flyamer I., Boyle S., Friman E. T. & Bickmore W. A. Transcription decouples estrogen-dependent changes in enhancer-promoter contact frequencies and physical proximity. 2023.03.29.534720 Preprint at 10.1101/2023.03.29.534720 (2023). PMC11152312 38781242

[33] Greca A. L. Higher-order chromatin organization defines Progesterone Receptor and PAX2 binding to regulate estradiol-primed endometrial cancer gene expression. bioRxiv 739466 (2020) doi:10.1101/739466.

[34] Rodriguez A. C. ETV4 Is Necessary for Estrogen Signaling and Growth in Endometrial Cancer Cells. Cancer Res. 80, 1234–1245 (2020). 32046982 10.1158/0008-5472.CAN-19-1382 PMC7073291

[35] Mumbach M. R. Enhancer connectome in primary human cells identifies target genes of disease-associated DNA elements. Nat. Genet. 49, 1602–1612 (2017). 28945252 10.1038/ng.3963 PMC5805393

[36] Carleton J. B., Berrett K. C. & Gertz J. Multiplex Enhancer Interference Reveals Collaborative Control of Gene Regulation by Estrogen Receptor α-Bound Enhancers. Cell Syst. 5, 333–344.e5 (2017). 28964699 10.1016/j.cels.2017.08.011 PMC5679353

[37] Zhou Y. Temporal dynamic reorganization of 3D chromatin architecture in hormone-induced breast cancer and endocrine resistance. Nat. Commun. 10, 1–14 (2019). 30944316 10.1038/s41467-019-09320-9 PMC6447566

[38] Gertz J. Distinct Properties of Cell-Type-Specific and Shared Transcription Factor Binding Sites. Mol. Cell 52, 25–36 (2013). 24076218 10.1016/j.molcel.2013.08.037 PMC3811135

[39] Rush C. M. Characterization of HCI-EC-23 a novel estrogen- and progesterone-responsive endometrial cancer cell line. Sci. Rep. 12, 19731 (2022). 36396974 10.1038/s41598-022-24211-8 PMC9672046

[40] Ginley-Hidinger M. Cis-regulatory control of transcriptional timing and noise in response to estrogen. Cell Genomics 4, 100542 (2024). 38663407 10.1016/j.xgen.2024.100542 PMC11099348

[41] Carleton J. B., Berrett K. C. & Gertz J. Dissection of Enhancer Function Using Multiplex CRISPR-based Enhancer Interference in Cell Lines. J. Vis. Exp. JoVE (2018) doi:10.3791/57883. PMC6101477 29912188

[42] Pollex T. Enhancer–promoter interactions become more instructive in the transition from cell-fate specification to tissue differentiation. Nat. Genet. 56, 686–696 (2024). 38467791 10.1038/s41588-024-01678-x PMC11018526

[43] Chen Z. Increased enhancer–promoter interactions during developmental enhancer activation in mammals. Nat. Genet. 56, 675–685 (2024). 38509385 10.1038/s41588-024-01681-2 PMC11203181

[44] Gu B. Transcription-coupled changes in nuclear mobility of mammalian cis-regulatory elements. Science 359, 1050–1055 (2018). 29371426 10.1126/science.aao3136 PMC6590518

[45] Luo H. LATS kinase–mediated CTCF phosphorylation and selective loss of genomic binding. Sci. Adv. 6, eaaw4651 (2020). 32128389 10.1126/sciadv.aaw4651 PMC7030924

[46] Witcher M. & Emerson B. M. Epigenetic Silencing of the p16INK4a Tumor Suppressor is Associated with Loss of CTCF Binding and a Chromatin Boundary. Mol. Cell 34, 271 (2009). 19450526 10.1016/j.molcel.2009.04.001 PMC2723750

[47] Pollex T. Chromatin gene-gene loops support the cross-regulation of genes with related function. Mol. Cell 84, 822–838.e8 (2024). 38157845 10.1016/j.molcel.2023.12.023

[48] Li G. Extensive Promoter-Centered Chromatin Interactions Provide a Topological Basis for Transcription Regulation. Cell 148, 84–98 (2012). 22265404 10.1016/j.cell.2011.12.014 PMC3339270

[49] Cho S. W. Promoter of lncRNA Gene PVT1 Is a Tumor-Suppressor DNA Boundary Element. Cell 173, 1398–1412.e22 (2018). 29731168 10.1016/j.cell.2018.03.068 PMC5984165

[50] Servant N. HiC-Pro: an optimized and flexible pipeline for Hi-C data processing. Genome Biol. 16, 259 (2015). 26619908 10.1186/s13059-015-0831-x PMC4665391

[51] Lareau C. A. & Aryee M. J. hichipper: a preprocessing pipeline for calling DNA loops from HiChIP data. Nat. Methods 15, 155–156 (2018). 29489746 10.1038/nmeth.4583 PMC10572103

[52] McLean C. Y. GREAT improves functional interpretation of cis-regulatory regions. Nat. Biotechnol. 28, 495–501 (2010). 20436461 10.1038/nbt.1630 PMC4840234

[53] CRISPR Guide RNA Design Tool | Benchling. https://www.benchling.com/crispr.

[54] Reddy T. E. Genomic determination of the glucocorticoid response reveals unexpected mechanisms of gene regulation. Genome Res. 19, 2163–2171 (2009). 19801529 10.1101/gr.097022.109 PMC2792167

[55] Langmead B., Trapnell C., Pop M. & Salzberg S. L. Ultrafast and memory-efficient alignment of short DNA sequences to the human genome. Genome Biol. 10, R25 (2009). 19261174 10.1186/gb-2009-10-3-r25 PMC2690996

[56] Li H. The Sequence Alignment/Map format and SAMtools. Bioinformatics 25, 2078–2079 (2009). 19505943 10.1093/bioinformatics/btp352 PMC2723002

[57] Zhang Y. Model-based Analysis of ChIP-Seq (MACS). Genome Biol. 9, R137 (2008). 18798982 10.1186/gb-2008-9-9-r137 PMC2592715

[58] Kursa M. B. & Rudnicki W. R. Feature Selection with the Boruta Package. J. Stat. Softw. 36, 1–13 (2010).

