# Supplementary material for: Estrogen-induced chromatin looping changes identify a subset of functional regulatory elements
Source: bioRxiv. 2025 Feb 4:2024.06.12.598690. Originally published 2024 Jun 14. Preprint. [Version 2] doi: 10.1101/2024.06.12.598690 (PMC11195280; doi:10.1101/2024.06.12.598690)
Supplement: Supplement 1 [file media-1.pdf]

Supplementary Information

Estrogen-induced chromatin looping changes identify a subset of functional regulatory elements

Hosiana Abewe[1,2], Alexandra Richey[1,3], Jeffery M Vahrenkamp[1,2], Matthew Ginley-Hidinger[1,3], Craig M Rush[1,2], Noel Kitchen[1,2], Xiaoyang Zhang[1,2,3], Jason Gertz[1,2,3,*]

1. Huntsman Cancer Institute, University of Utah, Salt Lake City, UT 84112, USA
2. Department of Oncological Sciences, University of Utah, Salt Lake City, UT 84112, USA
3. Department of Biomedical Engineering, University of Utah, Salt Lake City, UT 84112, USA

* Lead contact: jay.gertz@hci.utah.edu

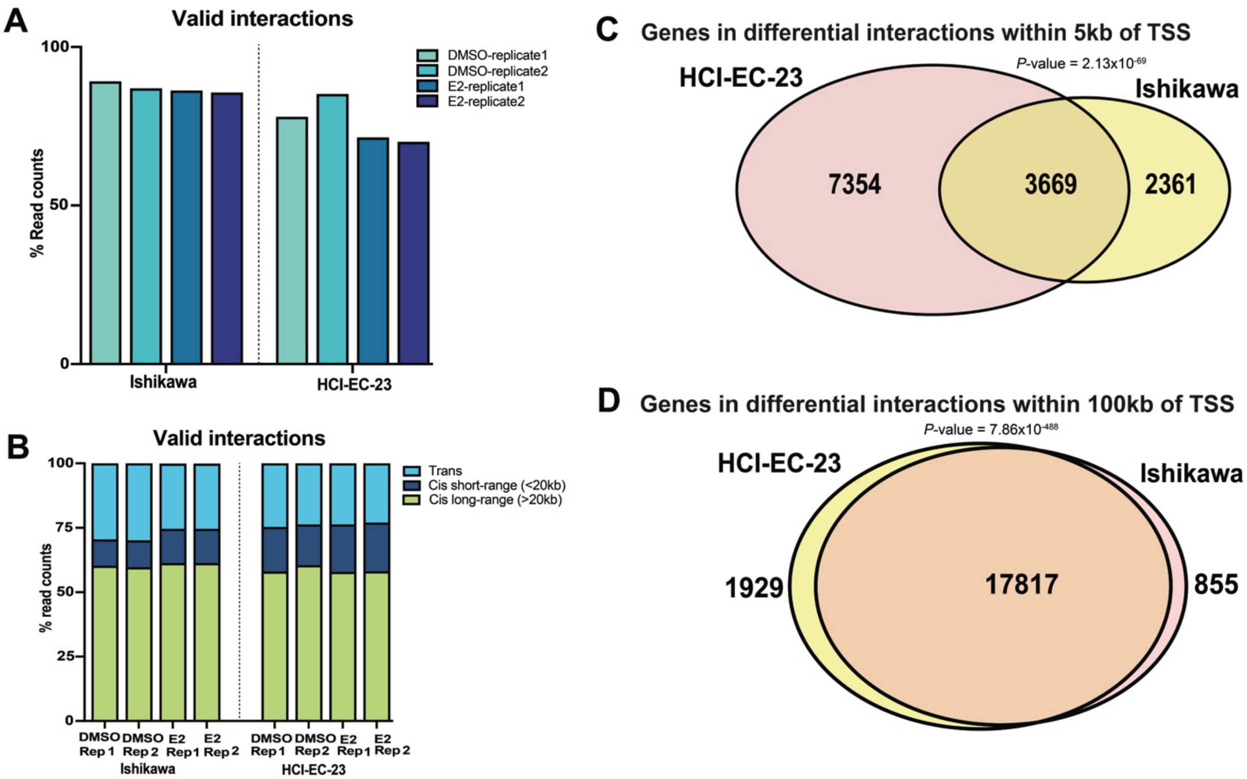

Supplementary Figure 1 (related to Figures 1 and 2). Quality control plots for HiChIP and diagram of genes with differential 3D interactions. A) Bar plot displays percentage of valid HiChIP reads in Ishikawa (left) and HCI-EC-23 (right) sample replicates. B) The distribution of trans (light blue), cis short-range (dark blue), or cis long-ranger (green) valid interactions in Ishikawa (left) and HCI-EC-23 (right) sample replicates is shown. (C-D) Venn diagrams display the overlap of genes associated with differential 3D interactions that have anchors within 5kb of TSS (C) or within 100kb of TSS (D) between HCI-EC-23 (yellow) and Ishikawa (pink).

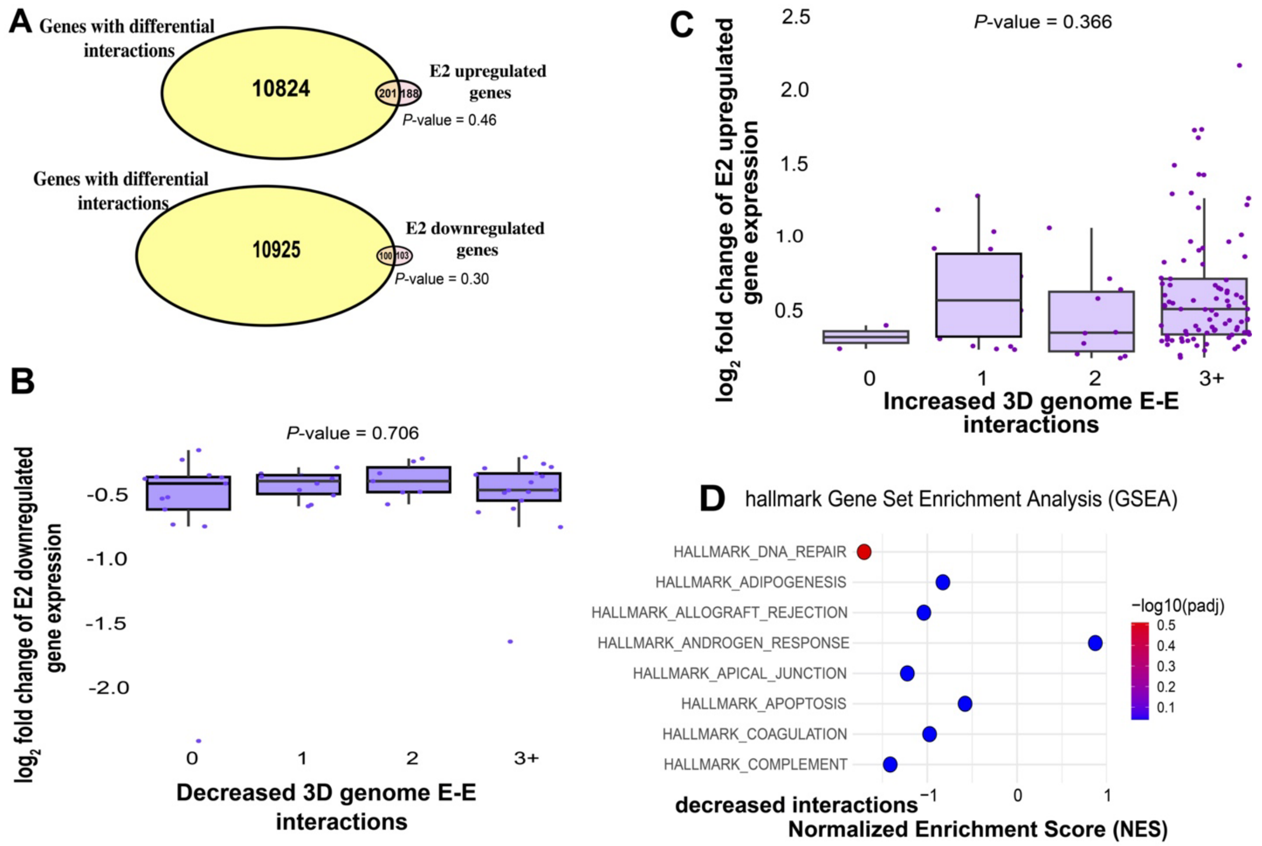

Supplementary Figure 2 (related to Figure 2). Comparison of genes associated with differential interactions and estrogen regulated genes. A) Overlap reveals E2 upregulated or downregulated genes (pink) that exhibit differential 3D interactions in HCI-EC-23 cells. (B-C) Boxplots demonstrate relative expression of E2 upregulated (B) and downregulated (C) genes associated with increasing (B) or decreasing (C) 3D genome interactions in HCI-EC-23 cells, respectively. P-values are from t-test on the linear regression coefficient . D) The GSEA rank of hallmark gene set enrichments based on the number of 3D genome interactions within 100 kb of each gene's TSS in HCI-EC-23 cells is shown.

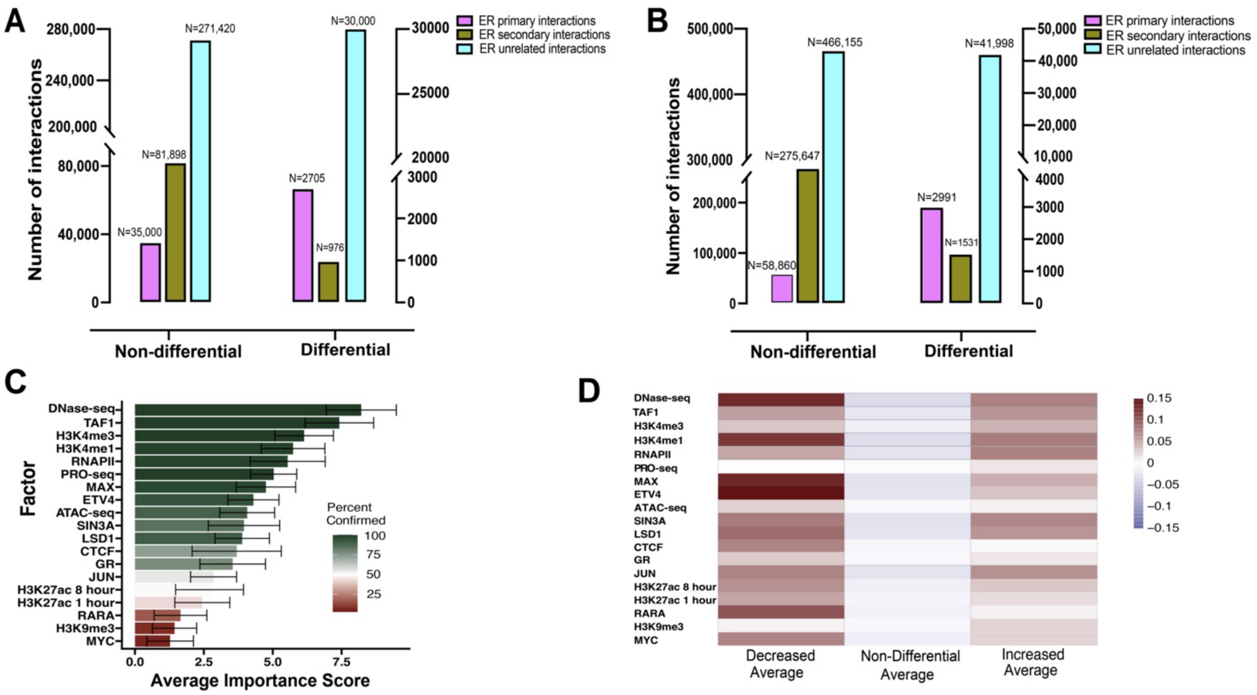

Supplementary Figure 3 (related to Figure 3). Characterization of 3D genome interactions associated with ER binding events. (A-B) The distribution of non-differential (left) or differential (right) 3D interaction anchors based on whether an anchor is an ERBS (magenta), an anchor interacts with an anchor that is an ERBS (green), or the two anchors of an interaction aren't involved in any loops that include an ERBS as an anchor (blue) is shown for Ishikawa (A) or HCI-EC-23 (B); N represents the number of 3D interactions for each category. C) The Boruta importance scores are shown for the analysis of anchors in differential compared to non-differential loops for ER-unrelated interactions. Only datasets collected in the absence of ER activation are shown. Error bars represent SD from 100 random down-sampled runs, and colors represent the percentage of time a variable is confirmed as important. D) Heatmap displays the average signal intensity for top-ranked genomic features from a Boruta analysis comparing ER-unrelated loops that are reduced, non-differential, or increased with E2 treatment in Ishikawa cells. Only datasets collected in the absence of ER activation are shown. Each feature's distributions were significantly different between at least one pair of groups based on a Kruskal Wallis test (False Discovery Rate < 0.05).

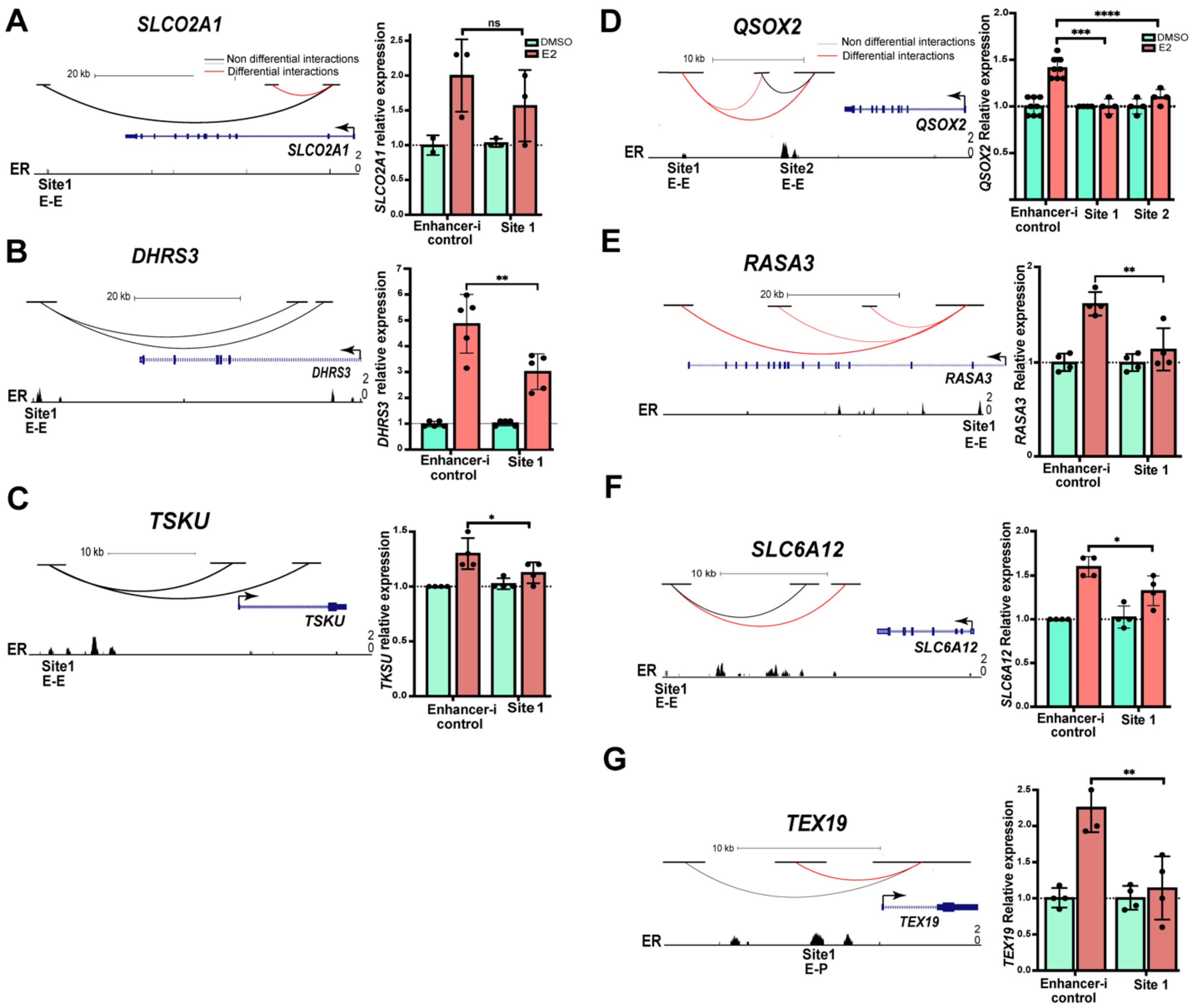

Supplementary Figure 4 (related to Figure 4). ERBS that serve as chromatin loop anchors are functionally important. (A-G) (left) Genome browser tracks of genes associated with ERBS with non-differential ((A) *SLCO2A1,*(B) *DHRS3,* (C) *TSKU*) and differential ((D) *QSOX2,* (E) *RASA3,* (F) *SLC6A12,* (G) *TEX19*) interactions are shown; red and black loops represent differential or non-differential interactions, respectively, and types of interactions are indicated by E-E or E-P under the browser track. ER ChIP-seq peaks are displayed at the bottom. (right) Relative expression of (A) *SLCO2A1,* (B) *DHRS3,* (C) *TSKU*, (D) *QSOX2,* (E) *RASA3,* (F) *SLC6A12,* and (G) *TEX19* are shown when an ERBS is targeted with Enhancer-i and cells are treated with DMSO (green) or E2 (orange). Error bars represent the SEM; ****p < 0.00001, ***p < 0.001, **p < 0.01 and *p < 0.05, unpaired t-test, while ns depict statistical insignificance.

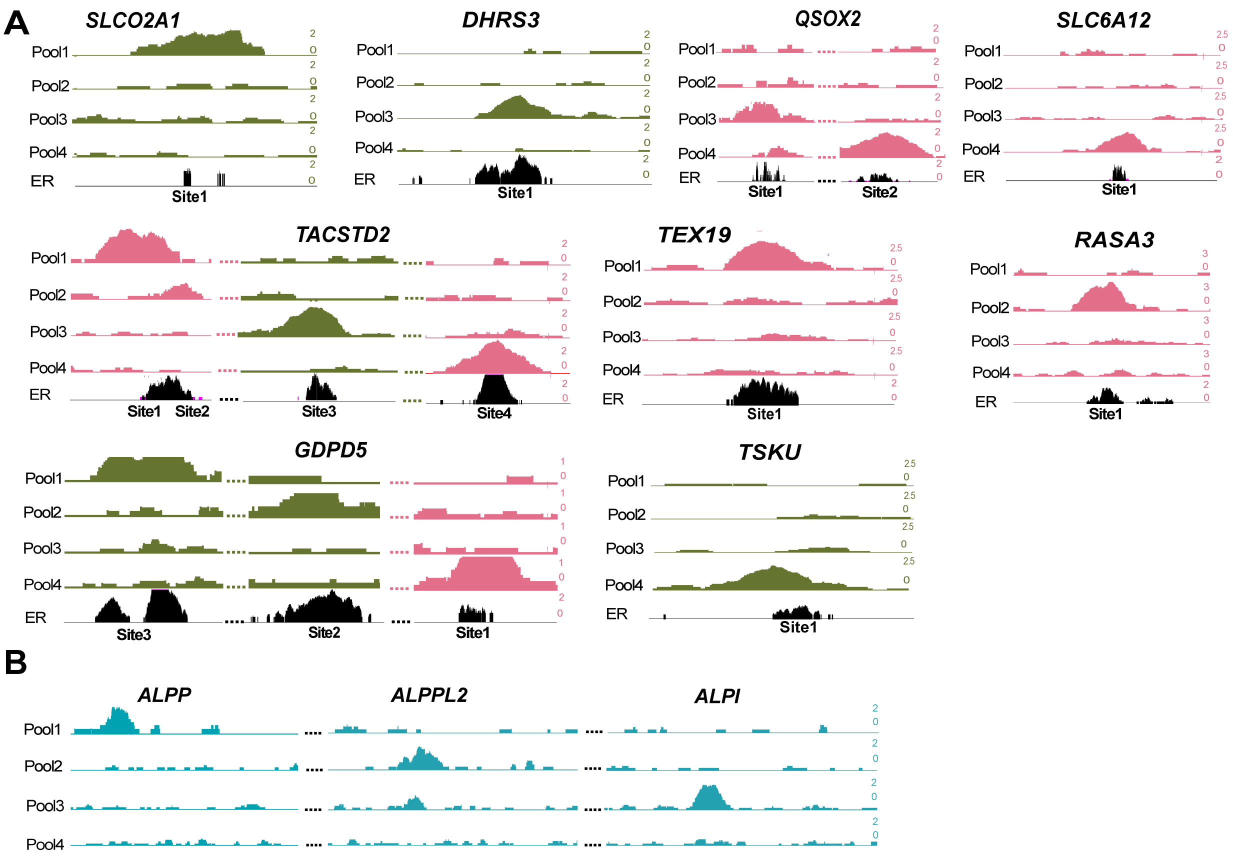

Supplementary Figure 5 (related to Figure 4 and 5). Enhancer-i successfully targets promoters and enhancers. A) ChIP-seq signal of Enhancer-i across pools of guide RNAs at targeted ERBS associated with differential (pink) or non-differential (green) interactions is shown along with ER ChIP-seq signal (black). B) ChIP-seq signal is shown for Enhancer-i across pools of guide RNAs at targeted promoters of genes that are part of an E2 upregulated gene cluster (*ALPP-APLPPL2-ALPI*).

Table S1. Total targeted ERBS

Site	Type of interaction		Functional	% reduction	Study
TEX19 - Site2	Promoter - Enhancer	Differential	Functional	100	This study
GDPD5 - Site1	Promoter - Enhancer	Differential	Functional	100	This study
GDPD5 - Site2	Enhancer-Enhancer	Non-differential	Functional	100	This study
GDPD5 - Site3	Enhancer-Enhancer	Non-differential	Functional	63	This study
MMP17 - Site3	Promoter - Enhancer	Differential	Non-functional	12.9	Carleton et al.
CISH - Site1	Promoter - Enhancer	Differential	Functional	44.8	Carleton et al.
CISH - Site2	Promoter - Enhancer	Differential	Functional	42.2	Carleton et al.
CISH - Site3	Promoter - Enhancer	Non-differential	Non-functional	3.9	Carleton et al.
FHL2 - Site1	Promoter - Enhancer	Non-differential	Functional	64.5	Carleton et al.
FHL2 - Site2	Promoter - Enhancer	Non-differential	Functional	42.5	Carleton et al.
FHL2 - Site3	Promoter - Enhancer	Non-differential	Non-functional	14.3	Carleton et al.
G0S2 - Site1	Promoter - Enhancer	Non-differential	Functional	93.3	Carleton et al.
MMP17 - Site1/2	Promoter - Enhancer	Non-differential	Functional	33.7	Carleton et al.
RASA3 - Site1	Enhancer-Enhancer	Differential	Functional	77.5	This study
QSOX2 - Site1	Enhancer-Enhancer	Differential	Functional	100	This study
QSOX2 - Site2	Enhancer-Enhancer	Differential	Functional	75.8	This study
SLC6A12 - Site2	Enhancer-Enhancer	Differential	Functional	50	This study
TACSTD2 - Site1	Enhancer-Enhancer	Differential	Non-functional	6.2	This study
TACSTD2 - Site2	Enhancer-Enhancer	Differential	Functional	51.9	This study
TACSTD2 - Site3	Enhancer-Enhancer	Non-differential	Non-functional	0	This study
TACSTD2 - Site4	Enhancer-Enhancer	Differential	Functional	88.3	This study
DHRS3 - Site1	Enhancer-Enhancer	Non-differential	Functional	48	This study
SLCO2A1 - Site1	Enhancer-Enhancer	Non-differential	Non-functional	46.7	This study
TKSU - Site1	Enhancer-Enhancer	Non-differential	Functional	66.7	This study

Table S2. gRNA sequences

gRNA name	gRNAs sequence	PAM
GDPD5 Enhancer gRNA 1	AGAGAGCAGGAAGCAAGTTG	GGG
GDPD5 Enhancer gRNA 2	CCAGTGTCCCCATATGACAT	AGG
GDPD5 Enhancer gRNA 3	GTTCTCCCTGTCATCCCAAG	AGG
GDPD5 Enhancer gRNA 4	GTAAACTGTACAGTGTGCAG	TGG
TEX19 Enhancer gRNA 1	TCTTACCTGTGTCCTCGGGA	TGG
TEX19 Enhancer gRNA 2	GAATGCCTCGCGCAGATCCG	GGG
TEX19 Enhancer gRNA 3	GGTGCTCAAAGAGCGTGACG	TGG
TEX19 Enhancer gRNA 4	TGCACCCCCGAGGTAGCTGG	GGG
RASA3 Enhancer gRNA 1	GAATAAGAAGGGCGATTCCA	GGG
RASA3 Enhancer gRNA 2	GCTCCCTGGCTATGGCATCG	TGG
RASA3 Enhancer gRNA 3	GCCAGGGCGCTCAGCACGAG	GGG
RASA3 Enhancer gRNA 4	GCTGCCATCCTAGCGCACAC	TGG
QSOX2 Enhancer 1 gRNA1	TTAGGTAACCAGAGGCTGAT	GGG
QSOX2 Enhancer 1 gRNA2	CCCCAAGAGCTCATGTGTCG	GGG
QSOX2 Enhancer 1 gRNA3	TATTCCCGGGACCGGACTGG	AGG
QSOX2 Enhancer 1 gRNA4	AGGTGCCAGGCCTTGGTCCG	AGG
QSOX2 Enhancer 2 gRNA1	CAGTGCCAGCGTATATGCGA	GGG
QSOX2 Enhancer 2 gRNA2	AGGCCAGGCCGACACCTCAA	TGG
QSOX2 Enhancer 2 gRNA3	CTGGTGAGGTCGACGCGCTG	TGG
QSOX2 Enhancer 2 gRNA4	CGCCTCTCCTGTTATCCGAG	GGG
SLC6A12 Enhancer 1 gRNA1	GGGAGAGGTGCCCATTCCCG	TGG
SLC6A12 Enhancer 1 gRNA2	AATCTCATGAGCTTTAGTGG	TGG
SLC6A12 Enhancer 1 gRNA3	TGGTTGGAGAGTGTTCCTCG	TGG
SLC6A12 Enhancer 1 gRNA4	TCACTCCAGCCGAGTCACAC	TGG
DHRS3 Enhancer gRNA1	ACCCCATCTAATACGCACCA	TGG
DHRS3 Enhancer gRNA2	GGGGATGTGGCCGTGAACTT	GGG
DHRS3 Enhancer gRNA3	AGTTCTTAAAGGCCAACTGT	AGG
DHRS3 Enhancer gRNA4	TTTTGTCCTCAAGGGGTAGC	AGG
TSKU Enhancer gRNA1	CCAGCACAGGTGAACTGAAT	GGG
TSKU Enhancer gRNA2	GTGCTACTCAGCACGAGCAG	AGG
TSKU Enhancer gRNA3	AAAGAGGAGGGCCCGATCGA	GGG
TSKU Enhancer gRNA4	GCAAAGCCAGCATTTGCTGA	GGG
SLCO2A1 Enhancer gRNA1	ACACTCAGACTAGGCAGCAT	GGG
SLCO2A1 Enhancer gRNA2	CTACCTGCTTCACTAGGAGT	CGG
SLCO2A1 Enhancer gRNA3	CAGAAACCCCCGTACTTGAC	CGG
SLCO2A1 Enhancer gRNA4	CACTGCACTTGGGTGTCCAA	GGG
ALPP Promoter gRNA1	AGGGCCCCAGCATGTCTGGA	GGG
ALPP Promoter gRNA2	TGTTGTGTAGGGGCAGCTCG	GGG
ALPP Promoter gRNA3	GCAGGGTCAAGGTGGCAACG	AGG
ALPP Promoter gRNA4	TCAATACCGCACCCTTCCCT	GGG
ALPPL2 Promoter gRNA1	ATGGTGGATGAACGAGTGAC	AGG
ALPPL2 Promoter gRNA2	AGCGGCGAGGCGGAAATCCC	AGG
ALPPL2 Promoter gRNA3	TGGCTCACTCACCGTCACCC	AGG
ALPPL2 Promoter gRNA4	CGACTGCTTCCAGACATGCA	GGG
ALPI Promoter gRNA1	GACATTACGAGCCCTAACCC	GGG

ALPI Promoter gRNA2	GGCACCCATATACACCAAGT	GGG
ALPI Promoter gRNA3	CGGGGTCAAGATGGACACCA	GGG
ALPI Promoter gRNA4	TCCTCCCCTGATTTAAACCC	AGG

Table S3. qPCR primers

Gene name	Forward sequence	Reverse sequence
CTCF	ACCTGTTCCTGTGACTGTACC	ATGGGTTCACTTTCCGCAAGG
GDPD5	CCCATGATGATACCACACCGT	CCAGCGATGTATGCGAAGG
TEX19	TCTACGCCTCCTGGATGTATC	CAGACCTGCATCTTCCAACCC
RASA3	AGATCAAGATCGGTGAAGCC	CTCCGTAAAACGGGCAGAGT
QSOX2	CTCGTGCAGTTCTACTCGTCG	AGCTCTCGGTCAGGTCCTTT
SLC6A12	GGCGGACCGTTTCTATGACA	AGGAGGGTGATGACGACGAA
TACSTD2	CGGCAGAACACGTCTCAGAAG	CCTTGATGTCCCTCTCGA AGTAG
DHRS3	TCTGTGATGTGGGCAACCG	ATGGTGATGTCACCCACCTTC
TSKU	TCCAGCCTAGCCAGTTTCTC	TGTTGTGCTGCTTGTGACA
SLCO2A1	GATGTCCACTGTCACCAAGC	GAATGACCTCCTTCGTTGGC
ALPP	CCCGCTTTAACCAGTGCAAC	GAGCTGCGTAGCGATGTCC
ALPPL2	GGCATCATCCCAGTTGAGGAG	GCACATGCTTGTCTACACTGTAT
ALPI	TACACGTCCATCCTGTACGG	CTCGCTCTCATTCACGTCTGG

Table S4. Datasets used in the Boruta analyses

Genomic Feature	Reference	Accession
ATAC	(Vahrenkamp et al. 2018)	GSE109890
CREB1	(ENCODE 2012)	GSE32465
CTCF	(ENCODE 2012)	GSE32465
DNase	(ENCODE 2012)	GSE32970
EGR1	(ENCODE 2012)	GSE32465
ER	(ENCODE 2012; Rodriguez et al. 2020)	GSE32465; GSE129803
ETV4	(ENCODE 2012)	GSE32465
FOXA1	(ENCODE 2012)	GSE32465
FOXM1	(ENCODE 2012)	GSE32465
GR	(Vahrenkamp et al. 2018)	GSE109893
H3K27ac	(Vahrenkamp et al. 2018)	GSE109893
H3K27me3	(Ginley-Hidinger et al. 2024)	GSE227241
H3K36me3	(Ginley-Hidinger et al. 2024)	GSE227241
H3K4me1	(Ginley-Hidinger et al. 2024)	GSE227241
H3K4me3	(Ginley-Hidinger et al. 2024)	GSE227241
H3K9me3	(Carleton et al. 2017)	GSE99906
JUN	(Ginley-Hidinger et al. 2024)	GSE227241
LSD1	(Ginley-Hidinger et al. 2024)	GSE227241
MAX	(Ginley-Hidinger et al. 2024)	GSE227241
MYC	(Ginley-Hidinger et al. 2024)	GSE227241
NFIC	(ENCODE 2012)	GSE32465
NRSF	(ENCODE 2012)	GSE32465
P300	(ENCODE 2012)	GSE32465
PRO-seq	(Ginley-Hidinger et al. 2024)	GSE227243
RAD21	(ENCODE 2012)	GSE32465
RARA	(Ginley-Hidinger et al. 2024)	GSE227241
RNAPII	(ENCODE 2012; Carleton et al. 2017)	GSE32465; GSE99906
SIN3A	(Ginley-Hidinger et al. 2024)	GSE227241
TAF1	(Ginley-Hidinger et al. 2024)	GSE227241
TCF12	(ENCODE 2012)	GSE32465
TEAD4	(ENCODE 2012)	GSE32465
USF1	(ENCODE 2012)	GSE32465
YY1	(ENCODE 2012)	GSE32465
ZBTB7A	(ENCODE 2012)	GSE32465

Supplemental References

Carleton JB, Berrett KC, Gertz J. 2017. Multiplex Enhancer Interference Reveals Collaborative Control of Gene Regulation by Estrogen Receptor α-Bound Enhancers. *Cell Syst* **5**: 333-344.e335.

ENCODE C. 2012. An integrated encyclopedia of DNA elements in the human genome. *Nature* **489**: 57-74.

Ginley-Hidinger M, Abewe H, Osborne K, Richey A, Kitchen N, Mortenson KL, Wissink EM, Lis J, Zhang X, Gertz J. 2024. Cis-regulatory control of transcriptional timing and noise in response to estrogen. *Cell Genom* **4**: 100542.

Rodriguez AC, Vahrenkamp JM, Berrett KC, Clark KA, Guillen KP, Scherer SD, Yang C-H, Welm BE, Janát-Amsbury MM, Graves BJ. 2020. ETV4 is necessary for estrogen signaling and growth in endometrial cancer cells. *Cancer research* **80**: 1234-1245.

Vahrenkamp JM, Yang CH, Rodriguez AC, Almomen A, Berrett KC, Trujillo AN, Guillen KP, Welm BE, Jarboe EA, Janat-Amsbury MM et al. 2018. Clinical and Genomic Crosstalk between Glucocorticoid Receptor and Estrogen Receptor α In Endometrial Cancer. *Cell Rep* **22**: 2995-3005.
