# Supplementary material for: Estrogen-induced chromatin looping changes identify a subset of functional regulatory elements
Source: bioRxiv. 2024 Jun 14:2024.06.12.598690. Preprint. [Version 1] doi: 10.1101/2024.06.12.598690 (PMC11195280; doi:10.1101/2024.06.12.598690)
Supplement: Supplementary file 1 [file media-1.pdf]

<u>**Supplementary Information**</u>

Estrogen-induced chromatin looping changes identify a subset of functional regulatory elements

Hosiana Abewe[1,2], Alexandra Richey[1,3], Jeffery M Vahrenkamp[1,2], Matthew Ginley-Hidinger[1,3], Craig M Rush[1,2], Noel Kitchen[1,2], Xiaoyang Zhang[1,2,3], Jason Gertz[1,2,3,*]

1. Huntsman Cancer Institute, University of Utah, Salt Lake City, UT 84112, USA
2. Department of Oncological Sciences, University of Utah, Salt Lake City, UT 84112, USA
3. Department of Biomedical Engineering, University of Utah, Salt Lake City, UT 84112, USA

* Lead contact: jay.gertz@hci.utah.edu

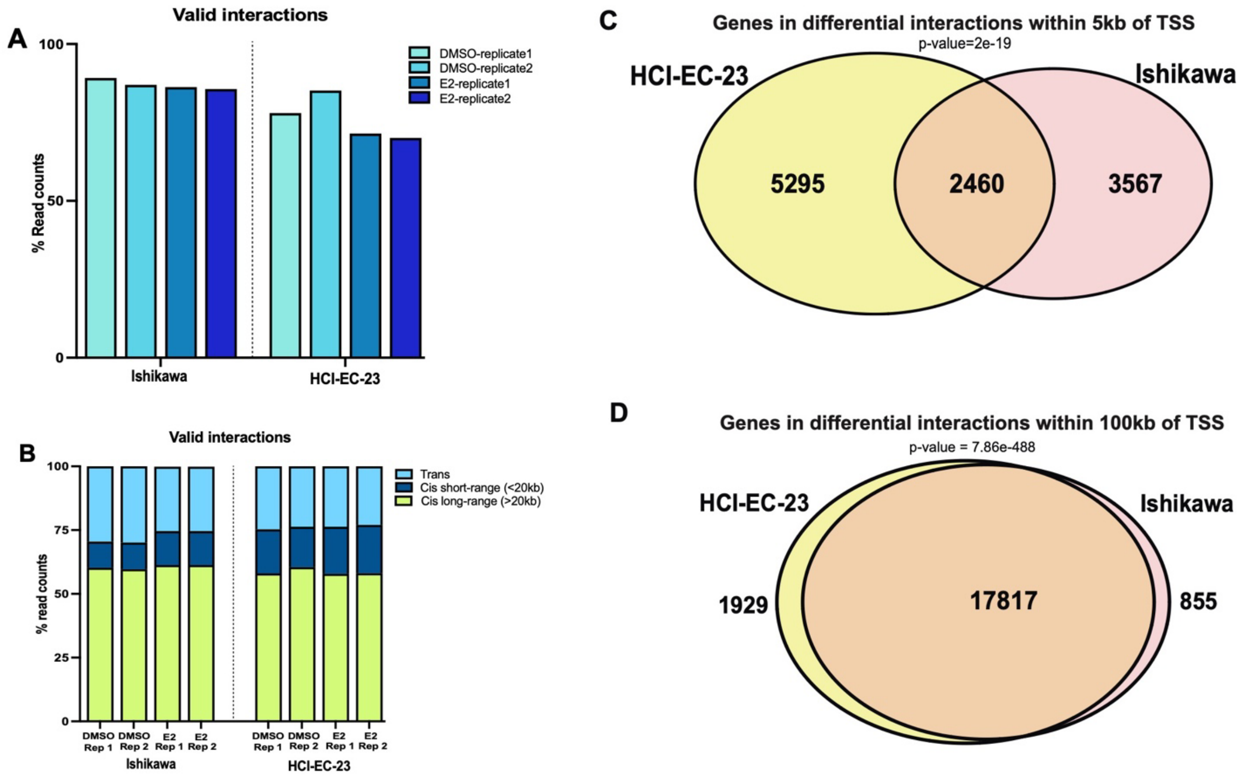

Supplementary Figure 1 (related to Figures 1 and 2). QC plots for HiChIP and diagram of genes with differential 3D interactions. A) Bar plot displays valid HiChIP reads in Ishikawa (left) and HCI-EC-23 (right) sample replicates. B) The distribution of trans (light blue), cis short-range (dark blue) , or cis long-ranger (green) valid interactions in Ishikawa (left) and HCI-EC-23 (right) sample replicates is shown. (C-D) Venn diagrams display the overlap of genes associated with differential 3D interactions that have anchors within 5kb of TSS (C) or within 100kb of TSS (D) between HCI-EC-23 (yellow) and Ishikawa (pink).

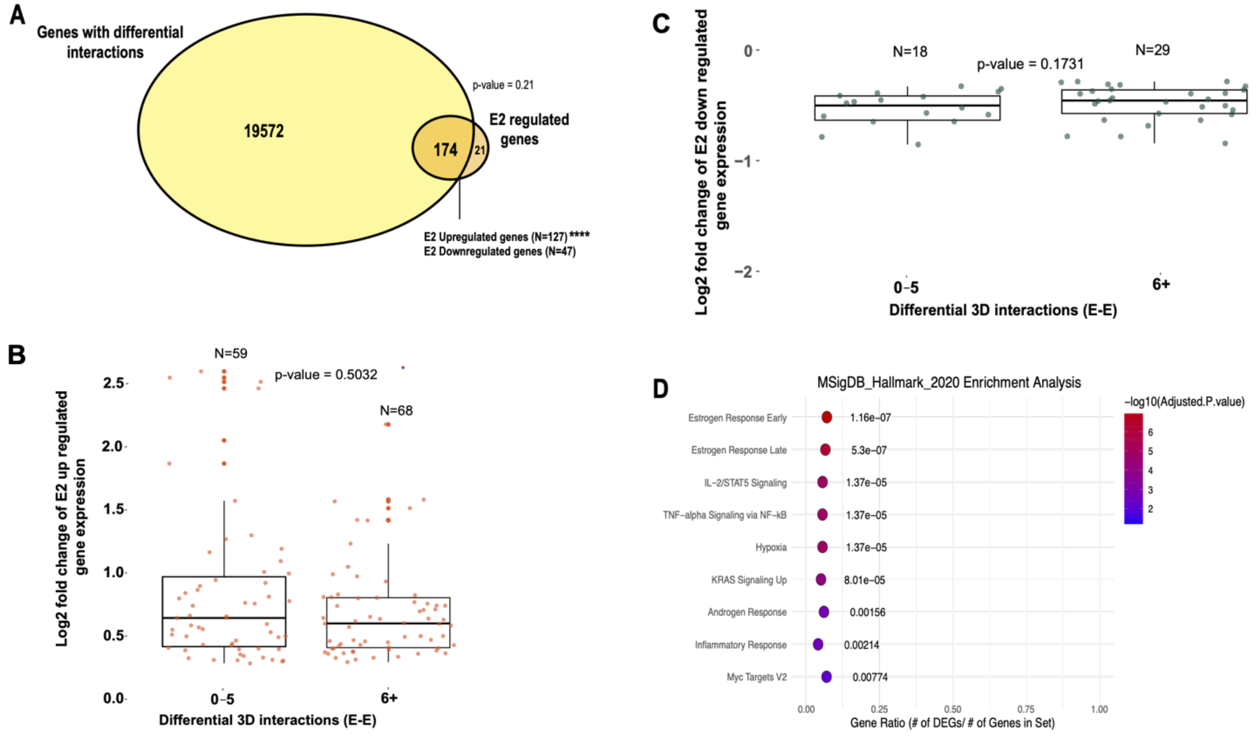

Supplementary Figure 2 (related to Figure 2). Comparison of genes associated with differential interactions and estrogen regulated genes. A) Overlap reveals E2 regulated genes that exhibit differential 3D interactions in HCI-EC-23 cells; ****p < 0.00001. (B-C) Boxplot demonstrates relative expression of E2 upregulated (B) and downregulated (C) genes associated with more than 5 differential 3D genome interactions or less than 6 differential 3D genome interactions in HCI-EC-23 cells. D) EnrichR ranking of gene ontology biological signaling pathways associated with E2 regulated genes with differential 3D genome interactions in HCI-EC-23 cells is shown.

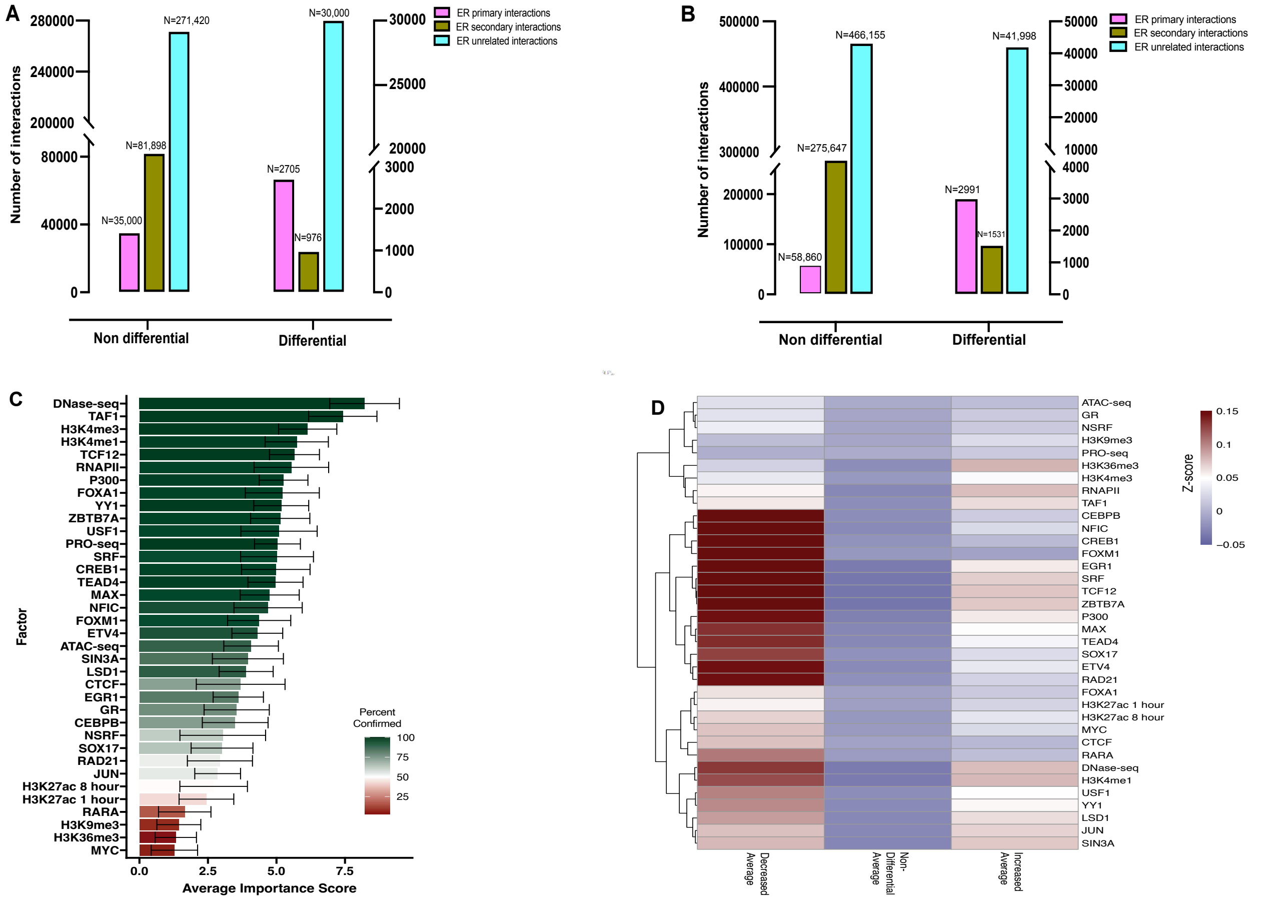

Supplementary Figure 3 (related to Figure 3). Characterization of 3D genome interactions associated with ER binding events. (A-B) The distribution of non-differential (left) or differential (right) 3D interactions' anchors based on whether an anchor is an ERBS (magenta), an anchor interacts with an anchor that is an ERBS through a different interaction (green), or the two anchors of an interaction aren't involved in any loops that include an ERBS as an anchor (blue) is shown for Ishikawa (A) or HCI-EC-23 (B); N represents the number of 3D interactions for each category. C) The Boruta importance scores are shown for the analysis of anchors in differential compared to non-differential loops. Error bars represent sd from 100 random down-sample runs and colors represent the percent of the time a variable is confirmed as important. D) Heatmap displays the average signal intensity for top-ranked genomic features from a Boruta analysis comparing loops that are reduced, non-differential or increased with E2 treatment in Ishikawa cells.

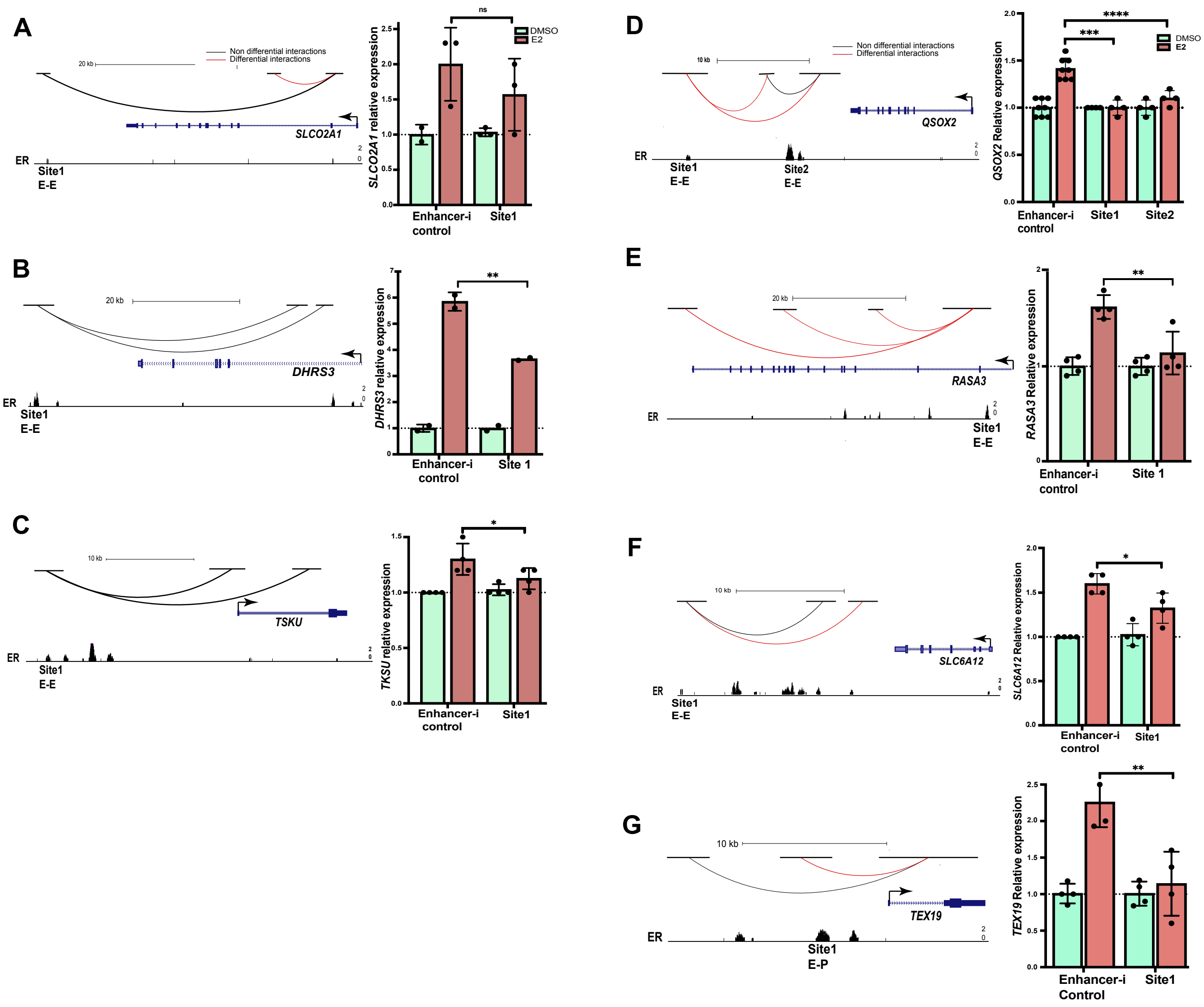

Supplementary Figure 4 (related to Figure 4). ERBS that serve as chromatin loop anchors are functionally important. (A-H) (left) Genome browser tracks of genes associated with ERBS with non-differential ((A) *SLCO2A1,*(B) *DHRS3,* (C) *TSKU*) and differential ((D) *QSOX2,* (E) *RASA3,* (F) *SLC6A12,* (G) *TEX19*) interactions are shown; red and black loops represent differential or nondifferential interactions, respectively, and types of interactions are indicated by E-E or E-P. ER ChIP-seq peaks are displayed at the bottom. (right) Relative expression of (A) *SLCO2A1,* (B) *DHRS3,* (C) *TSKU*, (D) *QSOX2,* (E) *RASA3,* (F) *SLC6A12,* (G) *TEX19* are shown when a candidate ERBS is targeted with Enhancer-i upon DMSO (green) or E2 (orange) treatment. Error bars represent the SEM; ****$p < 0.00001$, ***$p < 0.001$, **$p < 0.01$ and *$p < 0.05$, unpaired t-test, while ns depict statistical insignificance.

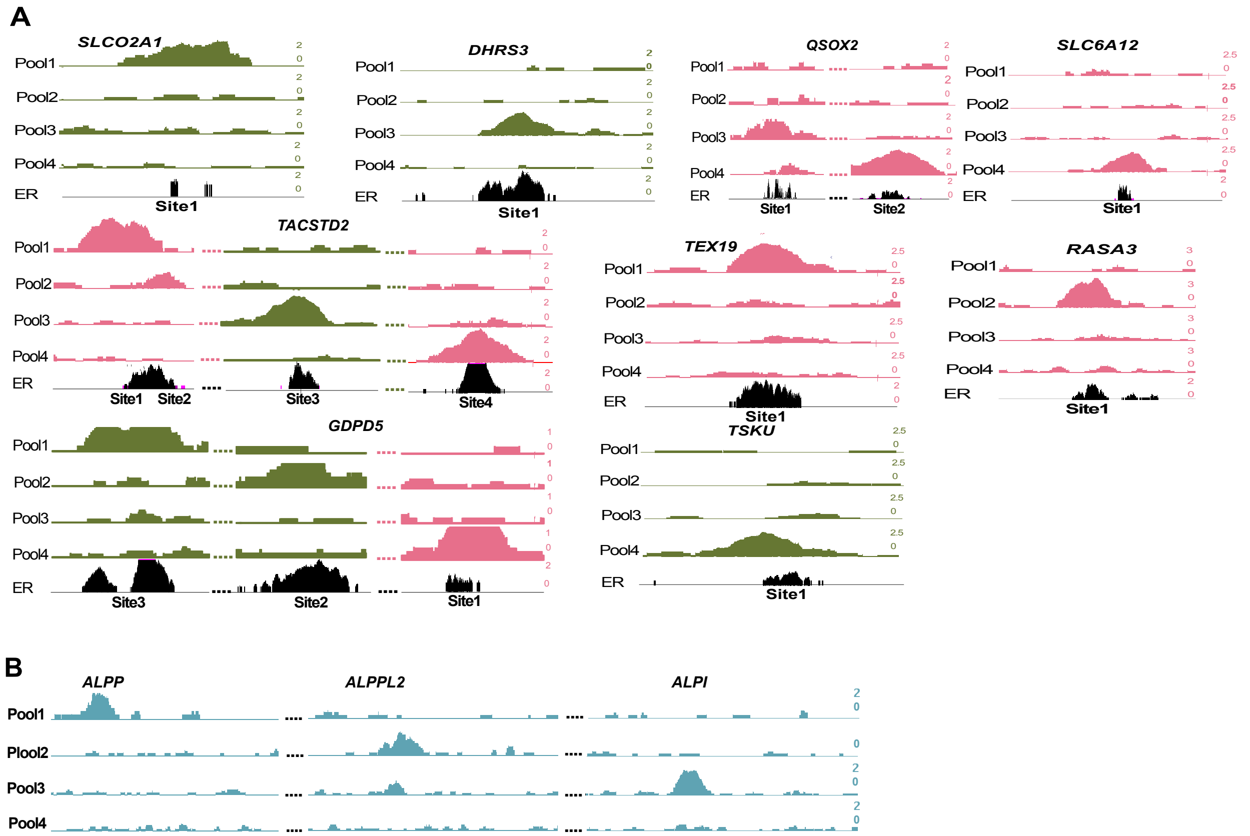

Supplementary Figure 5 (related to Figure 4 and 5). Enhancer-i successfully targets promoters and enhancers. A) ChIP-seq signal of Enhancer-i at targeted ERBS associated with differential (pink) or non-differential (green) interactions is shown along with ER ChIP-seq signal (black). B) ChIP-seq signal is shown for Enhancer-i promoter targeting of genes that are part of an E2 upregulated gene cluster (*ALPP-APLPPL2-ALPI*).

Table S1. gRNA sequences

gRNA NAME	gRNAs sequence	PAM
GDPD5 Enhancer gRNA 1	AGAGAGCAGGAAGCAAGTTG	GGG
GDPD5 Enhancer gRNA 2	CCAGTGTCCCCATATGACAT	AGG
GDPD5 Enhancer gRNA 3	GTTCTCCCTGTCATCCCAAG	AGG
GDPD5 Enhancer gRNA 4	GTAAACTGTACAGTGTGCAG	TGG
TEX19 Enhancer gRNA 1	TCTTACCTGTGTCCTCGGGA	TGG
TEX19 Enhancer gRNA 2	GAATGCCTCGCGCAGATCCG	GGG
TEX19 Enhancer gRNA 3	GGTGCTCAAAGAGCGTGACG	TGG
TEX19 Enhancer gRNA 4	TGCACCCCCGAGGTAGCTGG	GGG
RASA3 Enhancer gRNA 1	GAATAAGAAGGGCGATTCCA	GGG
RASA3 Enhancer gRNA 2	GCTCCCTGGCTATGGCATCG	TGG
RASA3 Enhancer gRNA 3	GCCAGGGCGCTCAGCACGAG	GGG
RASA3 Enhancer gRNA 4	GCTGCCATCCTAGCGCACAC	TGG
QSOX2 Enhancer 1 gRNA1	TTAGGTAACCAGAGGCTGAT	GGG
QSOX2 Enhancer 1 gRNA2	CCCCAAGAGCTCATGTGTCG	GGG
QSOX2 Enhancer 1 gRNA3	TATTCCCGGGACCGGACTGG	AGG
QSOX2 Enhancer 1 gRNA4	AGGTGCCAGGCCTTGGTCCG	AGG
QSOX2 Enhancer 2 gRNA1	CAGTGCCAGCGTATATGCGA	GGG
QSOX2 Enhancer 2 gRNA2	AGGCCAGGCCGACACCTCAA	TGG
QSOX2 Enhancer 2 gRNA3	CTGGTGAGGTCGACGCGCTG	TGG
QSOX2 Enhancer 2 gRNA4	CGCCTCTCCTGTTATCCGAG	GGG
SLC6A12 Enhancer 1 gRNA1	GGGAGAGGTGCCCATTCCCG	TGG
SLC6A12 Enhancer 1 gRNA2	AATCTCATGAGCTTTAGTGG	TGG
SLC6A12 Enhancer 1 gRNA3	TGGTTGGAGAGTGTTCCTCG	TGG
SLC6A12 Enhancer 1 gRNA4	TCACTCCAGCCGAGTCACAC	TGG
DHRS3 Enhancer gRNA1	ACCCCATCTAATACGCACCA	TGG
DHRS3 Enhancer gRNA2	GGGGATGTGGCCGTGAACTT	GGG
DHRS3 Enhancer gRNA3	AGTTCTTAAAGGCCAACTGT	AGG
DHRS3 Enhancer gRNA4	TTTTGTCCTCAAGGGGTAGC	AGG
TSKU Enhancer gRNA1	CCAGCACAGGTGAACTGAAT	GGG
TSKU Enhancer gRNA2	GTGCTACTCAGCACGAGCAG	AGG
TSKU Enhancer gRNA3	AAAGAGGAGGGCCCGATCGA	GGG
TSKU Enhancer gRNA4	GCAAAGCCAGCATTTGCTGA	GGG
SLCO2A1 Enhancer gRNA1	ACACTCAGACTAGGCAGCAT	GGG
SLCO2A1 Enhancer gRNA2	CTACCTGCTTCACTAGGAGT	CGG
SLCO2A1 Enhancer gRNA3	CAGAAACCCCCGTACTTGAC	CGG
SLCO2A1 Enhancer gRNA4	CACTGCACTTGGGTGTCCAA	GGG
ALPP Promoter gRNA1	AGGGCCCCAGCATGTCTGGA	GGG
ALPP Promoter gRNA2	TGTTGTGTAGGGGCAGCTCG	GGG
ALPP Promoter gRNA3	GCAGGGTCAAGGTGGCAACG	AGG
ALPP Promoter gRNA4	TCAATACCGCACCCTTCCCT	GGG
ALPPL2 Promoter gRNA1	ATGGTGGATGAACGAGTGAC	AGG
ALPPL2 Promoter gRNA2	AGCGGCGAGGCGGAAATCCC	AGG
ALPPL2 Promoter gRNA3	TGGCTCACTCACCGTCACCC	AGG
ALPPL2 Promoter gRNA4	CGACTGCTTCCAGACATGCA	GGG
ALPI Promoter gRNA1	GACATTACGAGCCCTAACCC	GGG

ALPI Promoter gRNA2	GGCACCCATATACACCAAGT	GGG
ALPI Promoter gRNA3	CGGGGTCAAGATGGACACCA	GGG
ALPI Promoter gRNA4	TCCTCCCCTGATTTAAACCC	AGG

Table S2. qPCR primers

GENE NAME	Forward sequence	Reverse sequence
CTCF	ACCTGTTCCTGTGACTGTACC	ATGGGTTCACTTTCCGCAAGG
GDPD5	CCCATGATGATACCACACCGT	CCAGCGATGTATGCGAAGG
TEX19	TCTACGCCTCCTGGATGTATC	CAGACCTGCATCTTCCAACCC
RASA3	AGATCAAGATCGGTGAAGCC	CTCCGTAAAACGGGCAGAGT
QSOX2	CTCGTGCAGTTCTACTCGTCG	AGCTCTCGGTCAGGTCCTTT
SLC6A12	GGCGGACCGTTTCTATGACA	AGGAGGGTGATGACGACGAA
TACSTD2	CGGCAGAACACGTCTCAGAAG	CCTTGATGTCCCTCTCGA AGTAG
DHRS3	TCTGTGATGTGGGCAACCG	ATGGTGATGTCACCCACCTTC
TSKU	TCCAGCCTAGCCAGTTTCTC	TGTTGTGCTGCTTGTGACA
SLCO2A1	GATGTCCACTGTCACCAAGC	GAATGACCTCCTTCGTTGGC
ALPP	CCCGCTTTAACCAGTGCAAC	GAGCTGCGTAGCGATGTCC
ALPPL2	GGCATCATCCCAGTTGAGGAG	GCACATGCTTGTCTACACTGTAT
ALPI	TACACGTCCATCCTGTACGG	CTCGCTCTCATTCACGTCTGG
